# Comparative Brain Imaging Reveals Analogous and Divergent Patterns of Species and Face Sensitivity in Humans and Dogs

**DOI:** 10.1523/JNEUROSCI.2800-19.2020

**Published:** 2020-10-21

**Authors:** Nóra Bunford, Raúl Hernández-Pérez, Eszter Borbála Farkas, Laura V. Cuaya, Dóra Szabó, Ádám György Szabó, Márta Gácsi, Ádám Miklósi, Attila Andics

**Affiliations:** ^1^Department of Ethology, Institute of Biology, Eötvös Loránd University, Budapest 1117, Hungary; ^2^Lendület Developmental and Translational Neuroscience Research Group, Institute of Cognitive Neuroscience and Psychology, Research Centre for Natural Sciences, Budapest 1117, Hungary; ^3^MTA-ELTE Lendület Neuroethology of Communication Research Group, Eötvös Loránd University, Budapest 1117, Hungary; ^4^Instituto de Neurobiología, Universidad Nacional Autónoma de México, Santiago de Querétaro 3001, Mexico; ^5^Department of Neuroradiology, Medical Imaging Centre, Semmelweis University, Budapest 1083, Hungary; ^6^MTA-ELTE Comparative Ethology Research Group, Budapest 1117, Hungary

**Keywords:** across-species representational similarity analysis, comparative neuroscience, conspecific-preference, dog, face-sensitivity, fMRI, visual processing

## Abstract

Conspecific-preference in social perception is evident for multiple sensory modalities and in many species. There is also a dedicated neural network for face processing in primates. However, the evolutionary origin and the relative role of neural species sensitivity and face sensitivity in visuo-social processing are largely unknown. In this comparative study, species sensitivity and face sensitivity to identical visual stimuli (videos of human and dog faces and occiputs) were examined using functional magnetic resonance imaging in dogs (*n* = 20; 45% female) and humans (*n* = 30; 50% female). In dogs, the bilateral mid suprasylvian gyrus showed conspecific-preference, no regions exhibited face-preference, and the majority of the visually-responsive cortex showed greater conspecific-preference than face-preference. In humans, conspecific-preferring regions (the right amygdala/hippocampus and the posterior superior temporal sulcus) also showed face-preference, and much of the visually-responsive cortex showed greater face-preference than conspecific-preference. Multivariate pattern analyses (MVPAs) identified species-sensitive regions in both species, but face-sensitive regions only in humans. Across-species representational similarity analyses (RSAs) revealed stronger correspondence between dog and human response patterns for distinguishing conspecific from heterospecific faces than other contrasts. Results unveil functional analogies in dog and human visuo-social processing of conspecificity but suggest that cortical specialization for face perception may not be ubiquitous across mammals.

**SIGNIFICANCE STATEMENT** To explore the evolutionary origins of human face-preference and its relationship to conspecific-preference, we conducted the first comparative and noninvasive visual neuroimaging study of a non-primate and a primate species, dogs and humans. Conspecific-preferring brain regions were observed in both species, but face-preferring brain regions were observed only in humans. In dogs, an overwhelming majority of visually-responsive cortex exhibited greater conspecific-preference than face-preference, whereas in humans, much of the visually-responsive cortex showed greater face-preference than conspecific-preference. Together, these findings unveil functional analogies and differences in the organizing principles of visuo-social processing across two phylogenetically distant mammal species.

## Abstract (precis)

Conspecific-preference in social perception is evident for multiple sensory modalities and in many species. There is also a dedicated neural network for face processing in primates. However, the evolutionary origin and the relative role of neural species sensitivity and face sensitivity in visuo-social processing are largely unknown.

## Introduction

Tuning to relevant classes of social stimuli is evidenced by both behavioral and neural processing preferences, but whether such preferences are because of comparable neural mechanisms across mammals remains equivocal. Conspecific-preference is reported in many species and across sensory modalities. Conspecific relative to heterospecific smells (Boulet et al., 2009; Guo et al., 2018) and vocalizations (Dooling et al., 1992; Belin et al., 2000; Petkov et al., 2008; Andics et al., 2014) elicit stronger behavioral and neural responses in multiple species. Visual conspecific-preference is also well-documented across mammals behaviorally (Pascalis and Bachevalier, 1998; da Costa et al., 2004; Dufour et al., 2006) but only in primates neurally (Blonder et al., 2004; Kriegeskorte et al., 2008b; Anzellotti and Caramazza, 2014; Minxha et al., 2017).

A visual processing preference that has received considerable empirical attention is face sensitivity. In primates, behavioral data implicate highly developed and specialized visual skills in facial information processing (Morton and Johnson, 1991; Valenza et al., 1996; Kanwisher et al., 1997; Cassia et al., 2004; Dufour et al., 2006). Imaging findings suggest that face processing in nonhuman primates (Tsao et al., 2003) and in humans (Duchaine and Yovel, 2015; Kanwisher et al., 1997) is supported by dedicated cortical patches/regions. The presence of non-conspecific-preferring face-sensitive regions in humans (Tong et al., 2000; Blonder et al., 2004) and non-conspecific-preferring face-sensitive neurons in macaques (Minxha et al., 2017) is further support of the potential centrality of faceness, in addition to or even beyond conspecificity, as an organizing principle for primate visual social perception.

In non-primate mammals, the role of faces in visuo-social perception is largely unknown. For navigating the environment, relative to primates, many non-primates rely less on vision, or rely more on non-facial visual cues (Leopold and Rhodes, 2010). Although to various non-primates faces are attractive stimuli, direct behavioral evidence for strictly-defined face sensitivity is scarce (Leopold and Rhodes, 2010). Up until most recently, neural face sensitivity has only been reported in sheep (Kendrick and Baldwin, 1987; Peirce et al., 2001).

Domestic dogs are an ideal test case for comparative investigations of non-primate face processing. Because of social proximity to humans, dogs have been a species of choice in comparative studies of social perception and, with recent advances in awake dog fMRI (Bunford et al., 2017), neural mechanisms thereof. Similarly to humans, dogs can differentiate conspecific from heterospecific visual stimuli (Racca et al., 2010). Furthermore, evidence indicates that dogs also rely on faces as an important source of information that is socially relevant (Gácsi et al., 2004) and that dogs are attracted to human faces and can differentiate familiar from novel human faces (Huber et al., 2013). Although prior data suggest that specific canine temporal regions respond more strongly to (human) faces than objects (Dilks et al., 2015; Cuaya et al., 2016), the designs of these small-sample fMRI studies do not allow for inferences about whether the observed sensitivity to (human) faces is driven by sensitivity to animacy or bodily stimuli in general, or to faceness in particular. Recent data show that some canine temporal regions respond more strongly to dog than human faces (Thompkins et al., 2018), but whether this conspecific-preference is face-specific remains untested. Others did not find any dog brain regions to respond more strongly to faces than scrambled images (Dilks et al., 2015; Szabó et al., 2020).

To comparatively assess the role of conspecificity and faceness in visuo-social perception beyond the primate order, here, we performed the same fMRI experiment in humans and dogs, using identical stimuli for both species: videos of human and dog faces and occiputs [i.e., back of the head, stringent comparison stimuli that are similar to faces in terms of animacy, familiarity (of the species to the viewer), intactness, and shape]. We hypothesized that (1) as in the auditory modality (Andics et al., 2014), conspecific-preference is a relevant organizing principle of visuo-social perception in both dog and human brains, and (2) face-preference is less central, relative to conspecific-preference, in dogs than in humans. To test these hypotheses, we conducted whole brain univariate and multivariate analyses, directly contrasted processing preferences in visually-responsive cortices of both species, and performed across-species representational similarity analyses (RSAs).

## Materials and Methods

### 

#### Participants

Data were collected in the context of a two-site (Hungary: Eötvös Loránd University and Mexico: Universidad Nacional Autónoma de México) project. Participants were 20 family dogs and 30 humans. Fourteen dogs were recruited from the Hungarian site and 6 were recruited from the Mexican site and all 30 humans were recruited from the Hungarian site. In Hungary, dog owners and humans were recruited through the Department of Ethology participant pool and website, popular social networking sites, and via snowball sampling and in Mexico, dog owners were recruited by research staff in dog parks and via snowball sampling. All procedures involving dogs met national and international guidelines for animal care and were approved by the appropriate ethics committees (the Food Chain Safety and Animal Health Directorate Government Office, Hungary, and the Bioethics Committee of the Institute of Neurobiology, Universidad Nacional Autónoma de México, Mexico). All procedures involving humans were approved by the appropriate ethics committee [Committee of Scientific and Research Ethics (ETT-TUKEB), Budapest, Hungary] and were in accordance with the 1964 Helsinki declaration and its later amendments. All humans participated voluntarily and provided written informed consent.

Dogs had an average age of 5.37 years (SD = 2.91, range = 2.5–11 years; five intact males, four intact females, six neutered males, five spayed females) and were all family dogs. Independent samples *t* tests indicated no cross-site differences in dogs' age or average number of scanning sessions needed (*p*s > 0.211). Humans had an average age of 32.3 years (SD = 7.5, range = 21–50 years). Most completed a master's degree or equivalent (47%), followed by bachelor's degree (37%), and high school degree (16%). Seven women and four men currently owned a dog and 12 women and 14 men had ever owned a dog. All participants had intact or corrected-to-intact vision and were free of major medical or neurologic illness as indicated by self-report. Exclusion criteria were contraindications to magnetic resonance imaging (e.g., claustrophobia, pregnancy, non-removable ferrous objects). No participants reported having experienced a traumatic experience with dogs.

#### Experimental design and procedure

Experimental and stimulus design were identical for dogs and humans. The experiment comprised six runs, each run containing 12 blocks, each block comprised of 4, 2-s long stimuli representing one of four conditions: dog face (DF), dog occiput (DO), human face (HF), and human occiput (HO). Each block was preceded by a 10-s window during which a fixation cross was presented, and during the preblock windows, participants were presented with a brief alerting sound via headphones. Stimulus order within blocks and block order within runs was pseudo-randomized so that within blocks 1–4, 5–8, or 9–12, there was not more than one block of the same condition, and so that across the 12 blocks, blocks of the same condition did not immediately follow one another. Participants received one of six randomizations. The total length of a run was 226 s. Stimuli were presented ∼155 cm in front of participants' eyes and controlled using MATLAB (version R2016a) Psychophysics Toolbox Version 3. Dogs were trained to lay motionless during scanning (Andics et al., 2014, 2016) and to look at the stimuli. Dogs viewed the presentation screen directly (on an MR compatible LCD Monitor NordicNeuroLab AS) in Hungary, and back-projected onto a white screen (using an Epson x14+ projector) in Mexico, while maintaining a sphinx position with their heads supported by a chinrest (Berns et al., 2013; Cuaya et al., 2016) and humans viewed the screen through a mirror attached to the head coil.

Dogs were tested in one run per session, with no more than four sessions per day and humans were tested in a single session. Sessions with dogs were continued until six functional runs (average number of days needed was 3.15, range 2–6) were obtained.

Sessions were continuously monitored (for dogs closing their eyes, or not being fixated at the stimuli for longer than 4 s) on a monitor by experimenters responsible for scanning participants (the first four authors). No scans had to be discarded for these reasons. Humans were instructed to passively view the stimuli.

#### fMRI stimuli

Stimuli consisted of color videos (with an approximate size of faces/occiputs from lowest point of the chin to highest point on top of the head = 28 cm) of unknown human and dog faces and human and dog occiputs (36 images of each), depicted in front of a uniform blue background (Movie 1).

Movie 1. fMRI stimuli. Video shows sample dynamic images, presented in color and dynamically for 2 s, representing each of four conditions human face, human occiput, dog face, and dog occiput. Stimulus design was identical for dogs and humans. 10.1523/JNEUROSCI.2800-19.2020.video.1

Movement in the videos involved minor facial movements, such as eyeblinks or subtle change in the exact direction or location of eye gaze, or minor head movements, such as vertical movement because of inhalation/exhalation. In case of human faces, individuals posed without glasses, jewelry, or intense make-up and with as neutral expression as possible. To avoid creating stimuli that are potentially threatening for dogs, human individuals were instructed not to look directly into the camera and similar dog footage was selected (i.e., with gaze slightly averted). In selecting our stimuli, we aimed to balance ecological validity, experimental control, and feasibility. Specifically, to enhance ecological validity and feasibility, we chose natural color images as it was essential that images are engaging and easily perceivable for dogs to ensure that they look at them during scanning. To ensure experimental rigor, recording of images was done under identical settings and circumstances (e.g., with regard to lighting and time of day) and differences in visual properties (brightness, contrast, hue, saturation, motion) were considered in analyses (see *Control tests for low-level visual property effects* below).

#### fMRI data acquisition and preprocessing

At both test sites, scanning was performed on a 3T MRI scanner (Ingenia 3T, Philips Medical System) using, for both dogs and humans, a BOLD-sensitive T2*-weighted echo-planar imaging sequence (both dogs and humans: TR = 3200 ms, TE = 29 ms, flip angle = 90°, 2.5-mm-thick slices with 0.5-mm gap; dogs: field of view: 300 × 198 × 110 mm, acquisition matrix 120 × 79; 37 axial slices; humans: field of view: 300 × 198 × 132 mm, acquisition matrix 120 × 79; 44 axial slices). Each of the six runs included 75 volumes. A high-resolution anatomic scan was also acquired at a separate session for dogs and at the end of the functional imaging session for humans, using a T1-weighted 3D TFE sequence, with 1 × 1 × 1 mm resolution with 180 slices, covering the whole brain, for anatomic localization.

For dogs at both sites, Philips SENSE coils and for humans a Philips dStream Head 32ch coil was used. The former at the Hungarian site consisted of two, 14 × 17 cm elliptical elements (Flex-M) and at the Mexican site of two 11-cm in diameter circular elements (Flex-S), with one placed under the dog's head and the other on top of the dog's head, fixed with plastic strips, as in previous studies (Andics et al., 2014, 2016).

Image preprocessing and statistical analysis were performed using SPM12 (https://www.fil.ion.ucl.ac.uk/spm) and followed conventional preprocessing steps (realignment, normalization to a preselected, individually labeled canine brain of an average-sized dog as template for dogs (Czeibert et al., 2019) and a Montreal Neurologic Institute (MNI) template for humans, resampling to 2 × 2 × 2 mm voxels, and smoothing with an isotropic Gaussian kernel (full-width at half-maximal 4 mm for dogs, 8 mm for humans)). For dogs, the longitudinal axis of the brain was established through the rostral and caudal commissures, and the origin for obtaining coordinates was set to the mid of the rostral commissure. Negative to positive *x*, *y*, and *z* coordinates are in millimeters and, as in MNI space for humans, denote left to right, posterior to anterior, and inferior to superior directions, respectively. For dogs and humans, if translation exceeded 3 mm, the scan was excluded (three dog runs and no human runs were affected by these criteria).

The average of maximal movements for each translation direction was below 2.059 mm for dogs (1.523 in the *x*, 0.947 in the *y*, and 2.059 in the *z* direction) and 0.605 mm for humans (0.183 in the *x*, 0.434 in the *y*, and 0.605 in the *z* direction) and, for each rotation axis, was below 1.196° (0.698 for pitch, 1.196 for roll, and 0.773 for yaw) and 0.571° (0.571 for pitch, 0.199 for roll, and 0.231 for yaw), respectively. The average of the maximum scan-to-scan movement per dog and per translation direction was 0.853 mm (0.730 in the *x*, 0.618 in the *y*, and 1.212 in the *z* direction) and per human and per direction was 0.212 mm (0.068 in the *x*, 0.277 in the *y*, and 0.289 in the *z* direction). The average of the maximum scan-to-scan movement per dog and per rotation axis was 0.416° (0.475 for pitch, 0.469 for roll, and 0.305 for yaw) and per human and per axis was 0.151° (0.281 for pitch, 0.077 for roll, and 0.095 for yaw).

#### fMRI data and statistical analysis

All statistical tests were two-tailed unless otherwise noted.

#### General linear model (GLM)

A general linear model was applied to the time series, convolved with the canonical hemodynamic response function and with a 128-s high-pass filter. Condition regressors were constructed for each condition, resulting in four regressors: DF, DO, HF, and HO, the effects of which were estimated for each voxel for each participant, with first level individual models also including movement correction parameters as nuisance regressors, and taken to the second level for whole-volume random effects analysis on the group level. Threshold for reporting for contrasts were *p* < 0.001 uncorrected and *p* < 0.05 cluster-corrected for familywise error (FWE) for dogs and *p* < 0.000001 uncorrected and *p* < 0.001 cluster-corrected for FWE for humans. To establish that findings are not a result of shortcomings of experimental design or stimuli, the overall level of visual responsiveness within the dog and human brain was examined in GLM analyses comparing all conditions to baseline (i.e., fixation cross; *p* < 0.001 uncorrected and *p* < 0.05 cluster-corrected for FWE).

To create a set of all face-sensitive and conspecific-sensitive regions for further characterization, first, we selected peaks from the F > O and conspecific (C) > heterospecific (He; i.e., H > D for humans, D > H for dogs) main contrasts, starting with the strongest peaks. Peaks closer than 16 mm to those already selected were skipped. Next, in case of unilateral response, specific contrasts were examined to determine whether a contralateral region can be identified. Two dog regions and eight human regions were thus identified and included in further analyses: for dogs, bilateral mid suprasylvian gyrus (mSSG) based on D > H and for humans, bilateral fusiform gyrus (FuG) and inferior occipital gyrus (IOG), right posterior middle temporal gyrus (pMTG), right anterior middle temporal gyrus (aMTG) and right amygdala/hippocampus (AMY), based on F > O, and left pMTG based on HF>HO (for data on all specific contrasts, see Extended Data Table 1-1).

To further characterize these regions, 2(F, O) × 2(H, D) × 2(left, right) ANOVAs (2 × 2 in case of unilateral activity) were conducted (interpreting only side main effects and interactions but not interpreting face and species main effects, to avoid double-dipping).

#### Control tests for low-level visual property effects

To assess whether observed differences in brain response were because of differences in visual properties or motion of the four stimulus categories, the parametric effects of the four visual properties (brightness, contrast, hue, and saturation) and motion of the experimental stimuli were tested in random effects parametric modulation analyses. First, to quantify each video's brightness, contrast, hue, and saturation, the value of each property on each frame was calculated and then averaged. The brightness, hue, and saturation of each pixel was calculated by converting it to its' representation in the HSL color representation, in which the appearance of a pixel is determined by a number value of these three components. The contrast of each image was defined as the standard deviation of the pixel intensities (root mean square contrast). The level of motion across consecutive frames was evaluated using the motion estimation functions of MATLAB's Computer Vision System Toolbox, and then averaged over the whole clip.

To this end, we first checked for differences across conditions in 2(F, O) × 2(H, D) ANOVAs. Then, low-level visual properties that emerged as significantly different in faceness contrasts were modeled as parametric modulators in face-sensitive regions and low-level visual properties that emerged as significantly different in conspecificity contrasts were modeled as parametric modulators in conspecific-preferring regions. Obtained mean β values were compared with zero, in a total of 23 Benjamini–Hochberg-corrected one-sample *t* tests, considering each of eight GLM-derived regions, the contrast based on which the region was identified, and whether or not there was a difference in any visual property for the pertinent comparison. For example, the bilateral IOG was selected based on F > O and, because faces and occiputs differed only in brightness, left and right IOG response to brightness was compared with zero. Accordingly, we tested R/L mSSG, R AMY, and R/L pMTG response to contrast, hue, and saturation, and R/L FuG, R/L IOG, R/L pMTG, R aMTG, and R AMY response to brightness.

As another test of the degree to which variations in visual properties modulated neural response, GLM analyses and then ANOVAs were repeated controlling for variations in visual properties, i.e., following removal of a single, visually most deviant block per condition, per run. To identify the visually most deviant block, we ranked all blocks within each condition and each run, giving the highest rank to the block which contributed the most to the visual difference across conditions. This ranking was done for all four visual properties across runs, and ranks were summed. For each condition and each run, the block with the highest rank was identified as the visually most deviant one. After removal of these deviant blocks, visual properties did not differ for the remaining trials, *p*s > 0.05.

#### Comparing conspecific-preference and face-preference

To examine the extent to which visually-responsive voxels respond stronger to the conspecificity or to the faceness of stimuli, first, the proportion of voxels with greater sensitivity to conspecificity than to faceness and the proportion with greater sensitivity to faceness than to conspecificity was assessed, by calculating (1) the number of voxels with larger positive β values in the C>He contrast at the group level than in the F > O contrast and (2) the number of voxels with larger positive β values in the F > O contrast at the group level than in the C>He contrast, respectively. Second, the proportion of these two sets of voxels was determined [a/(a + b)].

To assess the likelihood of obtaining the observed proportions by chance, we first modelled the proportion with greater sensitivity to conspecificity than to faceness and the proportion with greater sensitivity to faceness than to conspecificity under a “no signal” condition, by randomly re-labeling each stimulus block. Second, we determined the number of conspecific-preferring and face-preferring voxels and third, we employed permutation testing with 10,000 resamples.

To determine whether, across participants, there are sets of voxels exhibiting consistently greater conspecific-preference than face-preference (or vice versa), within the visually-responsive regions of each participant, a “response preference map” was created. A value of 1 was assigned to each voxel whose β value of the C>He contrast was positive and greater than the β value of the F > O contrast. A value of −1 was assigned to each voxel whose β value of the F > O contrast was positive and greater than the β value of the C>He contrast and a value of 0 was assigned to all other voxels. Then, the response preference map was compared with a mean of random permutations in one-sample *t* tests (one-tailed) at the group level, using SnPM. Thresholds for reporting for contrasts were *p* < 0.005 uncorrected and *p* < 0.05 cluster-corrected for FWE for dogs and *p* < 0.0001 uncorrected and *p* < 0.001 cluster-corrected for FWE for humans.

#### Multivariate pattern analysis (MVPA)

To assess which regions can accurately discriminate faces from occiputs (face sensitivity, F vs O) and conspecific from heterospecific stimuli (species sensitivity, C vs He) in each species, we performed MVPAs on stimulus blocks using PyMVPA software package (Hanke, 2009) and the LibSVM's implementation of the linear support vector machine (LSVM) classifier (www.csie.ntu.edu.tw/∼cjlin/libsvm/). Final processing was done using custom-made MATLAB scripts. The events in the time series of each acquisition were convolved to the hemodynamic response function, then each acquisition was linearly detrended and z-scored. A two-way classification was performed, wherein a LSVM classifier was trained with the time series values corresponding to the two stimulus categories for each analysis (either F vs O or C vs He). Classifier performance in each participant was evaluated using a leave-one-out cross-validation scheme, that is, all but one acquisitions were used to train the classifier (train), and the classifier predicted the stimulus category in the remaining acquisition (test). This process was repeated so that each acquisition was “test” once. Classifier performance was then calculated as the average number of correct classifications across participants and acquisitions.

We searched within the visually-responsive cortex using a searchlight approach (Kriegeskorte et al., 2006) and a spherical kernel. In each voxel within the visually responsive regions of each participant we created a sphere (radius = 4 mm for dogs and 8 mm for humans) and all the voxels contained within the sphere were used to train and test a LSVM classifier using a training and testing scheme identical to the one described above. The resulting classification accuracy was projected back to the center of the sphere. We repeated this process for every voxel, thus creating an accuracy map for each participant.

To determine whether classifier performance was better than chance, random permutation testing (Stelzer et al., 2013) was used. We calculated classifier performance that would be expected by chance for each voxel, by randomly re-labeling each stimulus block and repeating this process 10,000 times (to create a distribution of the possible values each voxel can have by chance) for dogs, and 1,000,000 times for humans. The probability of a given group mean classifier performance was then estimated, by comparing such performance to the performance that would be expected by chance. To test whether a region encoded information about a stimulus at the group level, we averaged the classification accuracy of each voxel across all participants. The resulting group map was then thresholded using permutation testing as described above (*p* < 0.001 for dogs and *p* < 0.000001 for humans). To estimate the probability of obtaining a cluster with a certain size, we used random permutation testing by repeating the same procedure. We then thresholded the obtained maps and calculated the number and size of clusters under chance conditions, and then used this distribution of cluster sizes to estimate the cluster size that would be expected by chance. Only clusters with sizes above threshold were retained (*p* < 0.05 for dogs and *p* < 0.001 for humans).

#### RSA

To assess whether stimuli are represented similarly in GLM-derived human brain regions and the dog brain, across-species RSAs (for a similar across-species comparison, see Kriegeskorte et al., 2008a) were performed, in multiple steps.

First, we calculated a representational dissimilarity matrix (RDM) for all stimulus categories across all runs of each participant. RDMs represent how different the patterns of activity are, related to a pair of stimuli, in a given set of voxels. For humans, we obtained RDMs for GLM-derived selected human peaks, creating a sphere (radius = 8 mm) around each peak. For dogs, we obtained RDMs using a searchlight approach (Connolly et al., 2012) by creating a sphere (radius = 4 mm) around each voxel in the visually-responsive cortex. (For completeness, we also report across-species representational similarities between the same human peaks and the whole dog brain in Extended Data Fig. 4-1.) RDMs were calculated as the correlation distance (1 – Pearson correlation) of each stimulus type-run pair of the activity pattern of the set of voxels within the sphere. To reduce differences between low-noise and high-noise voxels with regard to their impact, a transformation equivalent to univariate noise normalization suggested by Walther et al. (2016) was implemented. Specifically, before calculation of RDMs, the data of each voxel were rescaled, using the SD of changes in its “activation” during baseline periods.

Second, we compared human RDMs to dog RDMs. Two ways of across-species matching of conditions were tested. (1) Direct matching: human representations of human stimuli were compared with dog representations of human stimuli, and human representations of dog stimuli were compared with dog representations of dog stimuli. (2) Functional matching: human representations of human stimuli were compared with dog representations of dog stimuli, and human representations of dog stimuli were compared with dog representations of human stimuli. Direct matching therefore referenced stimulus identity, while functional matching referenced conspecificity/heterospecificity. We calculated Pearson correlation coefficients between RDMs, repeated this procedure for each voxel, and projected back the result of the correlation to the center of the sphere, obtaining a similarity map. We repeated this procedure for all dog-human pairs and averaged the maps of each human. A one-tailed one sample *t* test was run on each voxel at the group level (*p* < 0.001) to test whether the values of the voxel differed from chance (calculated by taking random coordinates and performing the same procedure, *n* = 1000).

Third, in cases where suprathreshold representational similarity across species was observed (this happened only for functional matching), to determine what is driving that similarity, follow-up pairwise comparisons were calculated in one sample *t* tests, comparing observed mean ρ values to expected (by chance) mean ρ values. (To obtain mean ρ values, correlation coefficients were calculated for every stimulus pair for each human*dog pair and then the means of these correlation coefficients for every stimulus pair were calculated for each participant; to obtain expected mean ρ values, the same procedure as for observed mean ρs was followed, except we randomly swapped condition labels, thereby obtained a chance mean ρ, repeated this 10,000 times and calculated their mean.) Comparisons of stimulus pairs CF versus CO (indicative of face sensitivity for conspecifics), HeF versus HeO (face sensitivity for heterospecifics), and HeF versus CF (species sensitivity for faces), HeO versus CO (species sensitivity for occiputs) were performed. To determine the magnitude of the obtained differences, Cohen's *d* values as indices of effect size were calculated for each pair compared.

#### Data availability

The datasets generated and/or analyzed during the current study are available from a corresponding author on reasonable request.

## Results

### GLM

**Figure 1.**
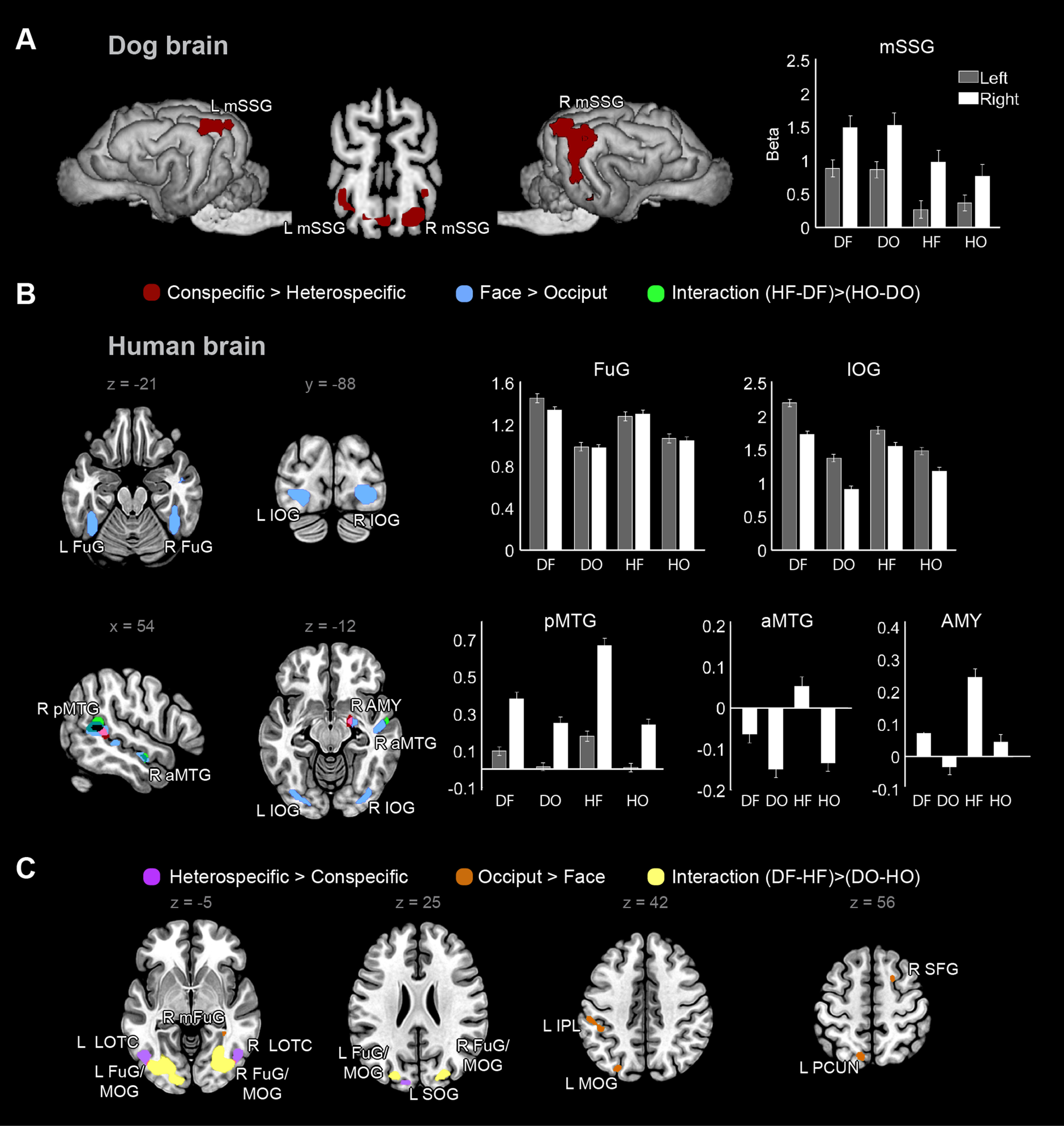
GLM results in dogs (*n* = 20) and humans (*n* = 30). ***A***, Dog contrast maps superimposed on a template brain (Czeibert et al., 2019). Threshold was *p* < 0.001 uncorrected and *p* < 0.05 cluster-corrected for FWE. None of the other main or interaction contrasts yielded significant effects. The bar graph represents parameter estimates (β weights) in select GLM-derived peaks (sphere radius = 4 mm) to each condition; error bars represent SE. ***B***, ***C***, Human contrast maps (main and interaction effects) superimposed on a template brain. Threshold was *p* < 0.000001 uncorrected and *p* < 0.001 cluster-corrected for FWE. ***B***, Conspecific>heterospecific, face>occiput, and their interaction. The bar graphs represent parameter estimates (β weights) in select GLM-derived peaks (sphere radius = 8 mm) to each condition; error bars represent SE. ***C***, Heterospecific>conspecific, occiput>face, and their interaction. D = dog; H = human; F = face; O = occiput; L = left; R = right; mSSG = mid suprasylvian gyrus; AMY = amygdala/hippocampus; aMTG = anterior middle temporal gyrus; FuG = fusiform gyrus; FuG/MOG = a cluster including parts of FuG, IOG, MOG and SOG; IOG = inferior occipital gyrus; IPL = inferior parietal lobule; LOTC = lateral occipitotemporal cortex; mFuG = medial fusiform gyrus; MOG = middle occipital gyrus; PCUN = precuneus; pMTG = posterior middle temporal gyrus; SFG = superior frontal gyrus; SOG = superior occipital gyrus, extending to cuneus.

For GLM results for each main contrast (F > O, O > F, H > D, D > H) and interactions in dogs and humans, see Table 1 and Figure 1. For visual responsiveness results in dogs and humans, see Extended Data Figures 2-1, 2-2.

**Table 1.** Main GLM results for dogs and humans

	Brain region	Cluster size (voxels)	Peak T	Coordinates (*x*, *y*, *z*)
Dogs				
D > H	**R mSSG**^a^	347	6.600	**14, −32, 22**
	**L mSSG**^a^		4.964	**−16, −22, 20**
Humans, main effects				
F > O	**R aMTG**	180	10.318	**48, −12, −14**
	**R IOG**	398	10.262	**28, −92, −2**
	**L IOG**	410	9.932	**−38, −82, −10**
	**R pMTG**	307	9.407	**52, −50, 10**
	**L FuG**	230	8.984	**−42, −54, −22**
	**R FuG**	235	8.952	**42, −46, −20**
	**R AMY**	56	8.260	**22, −6, −12**
	**L pMTG**^b^	51	7.520	**−50, −46, 12**
O > F	L IPL	122	8.279	−54, −30, 42
	L MOG	65	7.942	−28, −78, 42
	R SFG	44	7.595	22, 10, 58
	R mFuG	83	6.914	30, −52, −2
	L PCUN	81	6.824	−10, −68, 56
H > D	R pMTG	197	8.110	50, −40, 6
	R AMY	77	7.745	18, −12, −16
D > H	L LOTC	251	8.537	−52, −68, −4
	R LOTC	204	7.817	44, −62, 2
	L SOG	47	7.755	−8, −92, 24
Humans, interaction effects				
HF-DF>HO-DO	R pMTG	210	8.508	52, −44, 16
	R aMTG	33	7.691	56, −8, −14
DF-HF>DO-HO	L FuG/MOG	2562	12.093	−32, −86, 14
	R FuG/MOG	2045	9.741	24, −70, −16

Threshold for reporting for all higher-level contrasts was *p* < 0.000001 and cluster *p* < 0.001 for FWE for humans and *p* < 0.001 and cluster *p* < 0.05 for FWE for dogs. All peaks ≥16 mm apart are reported.

*^a^*At *p* < 0.001, these two peaks result from D > H as a single cluster's two main peaks. When checked with a stricter *p* < 0.0005 threshold, a left and a right cluster-corrected significant cluster is obtained, with the same peaks. Thus, in dogs, the main and the subpeak are reported but in humans, in the absence of single bilateral clusters, subpeaks are not reported.

*^b^*Region identified based on HF>HO.

L = left; R = right; mSSG = mid suprasylvian gyrus; aMTG = anterior middle temporal gyrus; IOG = inferior occipital gyrus; pMTG = posterior middle temporal gyrus; FuG = fusiform gyrus; AMY = amygdala/hippocampus; IPL = inferior parietal lobule; MOG = middle occipital gyrus; SFG = superior frontal gyrus; mFuG = medial fusiform gyrus; PCUN = precuneus; LOTC = lateral occipitotemporal cortex; SOG = superior occipital gyrus/cuneus; FuG/MOG = a cluster including parts of FuG, IOG, MOG, and SOG. Selected conspecific-preferring and face-sensitive regions are in bold. See also Extended Data Tables 1-1, 1-2, 1-3, 1-4.

10.1523/JNEUROSCI.2800-19.2020.t1-1 Table 1-1 Extended GLM results for dogs and humans. Download Table 1-1, DOCX file

10.1523/JNEUROSCI.2800-19.2020.t1-2 Table 1-2 Parametric modulation effects of basic visual properties (uncorrected). Download Table 1-2, DOCX file

10.1523/JNEUROSCI.2800-19.2020.t1-3 Table 1-3 ANOVA face and species main effects with all stimuli and with visually most deviant stimulus blocks excluded. Download Table 1-3, DOCX file

10.1523/JNEUROSCI.2800-19.2020.t1-4 Table 1-4 Individual differences with regard to breed, human face-related experience, and cephalic index across dogs. Download Table 1-4, DOCX file

In dogs, we found significant main effects only for the D > H contrast. Specifically, the bilateral mSSG responded more strongly to dog relative to human stimuli. Even with a more liberal, *p* < 0.005 uncorrected voxel threshold, we obtained no face-preferring >3-voxel clusters, *p*s(cluster-corrected for FWE) > 0.991 for 1–3-voxel clusters. In dogs, we found no interaction effects.

In humans, we found significant main effects for all four contrasts, with H > D regions essentially being a subset of F > O regions. Specifically, the bilateral FuG and IOG, right pMTG, right aMTG, and right AMY responded more strongly to faces relative to occiputs. Both the right pMTG and the right AMY responded more strongly to human than to dog stimuli. In the left hemisphere, the middle occipital gyrus (MOG), precuneus (PCUN), and inferior parietal lobule (IPL) and in the right hemisphere a medial FuG region (mFuG) and the superior frontal gyrus (SFG) responded more strongly to occiputs than to faces; and the left superior occipital region spanning to the cuneus (SOG) and bilateral lateral occipitotemporal cortex (LOTC) showed stronger response to dog than to human stimuli. In humans, we also found interaction effects: in the right pMTG and aMTG, there was stronger face-preference for conspecifics than heterospecifics. Follow-up comparisons indicated that response was greatest to human faces relative to all other stimuli (pMTG *p*s < 0.007, aMTG *p*s < 0.001), with no response difference among the other three conditions (pMTG *p*s > 0.877, aMTG *p*s > 0.993). This reveals conspecific face sensitivity in the right pMTG and aMTG. In the bilateral FuG/MOG, response was weaker to human faces than to either dog faces (L *p* = 0.012, R *p* = 0.071) or human occiputs (L *p* = 0.033, R *p* = 0.094), with no difference among other conditions (L *p*s > 0.129, R *p*s > 0.500).

Activity response profiles for selected GLM-derived regions in dogs and humans are shown in Figure 1*A*,*B*.

Further characterizing these regions, in dogs, for mSSG, neither the side main effect, nor any of the two-way or three-way interactions were significant (all *p*s > 0.164). In humans, for IOG, the main effect of side was significant, *F*_(1,239)_ = 20.286, *p* < 0.001 (left>right), and so was the interaction effect between face and species on IOG response, *F*_(1,239)_ = 8.530, *p* = 0.004, with greatest IOG response to dog faces. For FuG, neither the main effect of side, nor any of the two-way or three-way interactions were significant (all *p*s > 0.092). For pMTG, the main effect of side was significant, *F*_(1,239)_ = 66.947, *p* < 0.001 (right>left). Interactions between face and species (*F*_(1,239)_ = 6.396, *p* = 0.012) and face and side (*F*_(1,239)_ = 4.073, *p* = 0.045) were also significant. In case of the face by species interaction, greatest pMTG response was to human faces. In case of the face by side interaction, greatest pMTG response was to faces in the right hemisphere. For right AMY and right aMTG, the face by species interactions were not significant (*p* = 0.079 and *p* = 0.053, respectively).

### Control tests for low-level visual property effects

2(F, O) × 2(H, D) ANOVAs indicated a visual difference for four properties: for F > O, there was a difference in brightness *F*_(1,144)_ = 6.187, *p* = 0.014; but not hue, contrast, or saturation (all *p*s > 0.404). For H > D, there was a difference in contrast, *F*_(1,144)_ = 8.334, *p* = 0.004; hue, *F*_(1,144)_ = 4.007, *p* = 0.047; and saturation, *F*_(1,144)_ = 7.252, *p* = 0.008. There was no difference in motion (both *p*s > 0.353).

One-sample *t* tests indicated three cases with visual effects, all for humans: brightness contributed with a negative parametric modulatory effect to the right IOG response, *t*_(29)_ = −3.588, *p* = 0.001 (faces had greater brightness than occiputs), contrast contributed with a positive parametric modulatory effect to the right pMTG response, *t*_(29)_ = 3.453, *p* = 0.001 (human stimuli had greater contrast than dog stimuli), and brightness contributed with a positive parametric modulatory effect to the right pMTG response, *t*_(29)_ = 3.301, *p* = 0.002 (face stimuli had greater brightness than occiput stimuli; Extended Data Table 1-2).

When GLM analyses and then ANOVAs were repeated following removal of a single, visually most deviant block per condition, there were no changes in face or species main effects in any of the selected regions: all previously significant effects remained significant and no nonsignificant face or species main effect emerged as significant (Extended Data Table 1-3).

### Comparing conspecific-preference and face-preference

**Figure 2.**
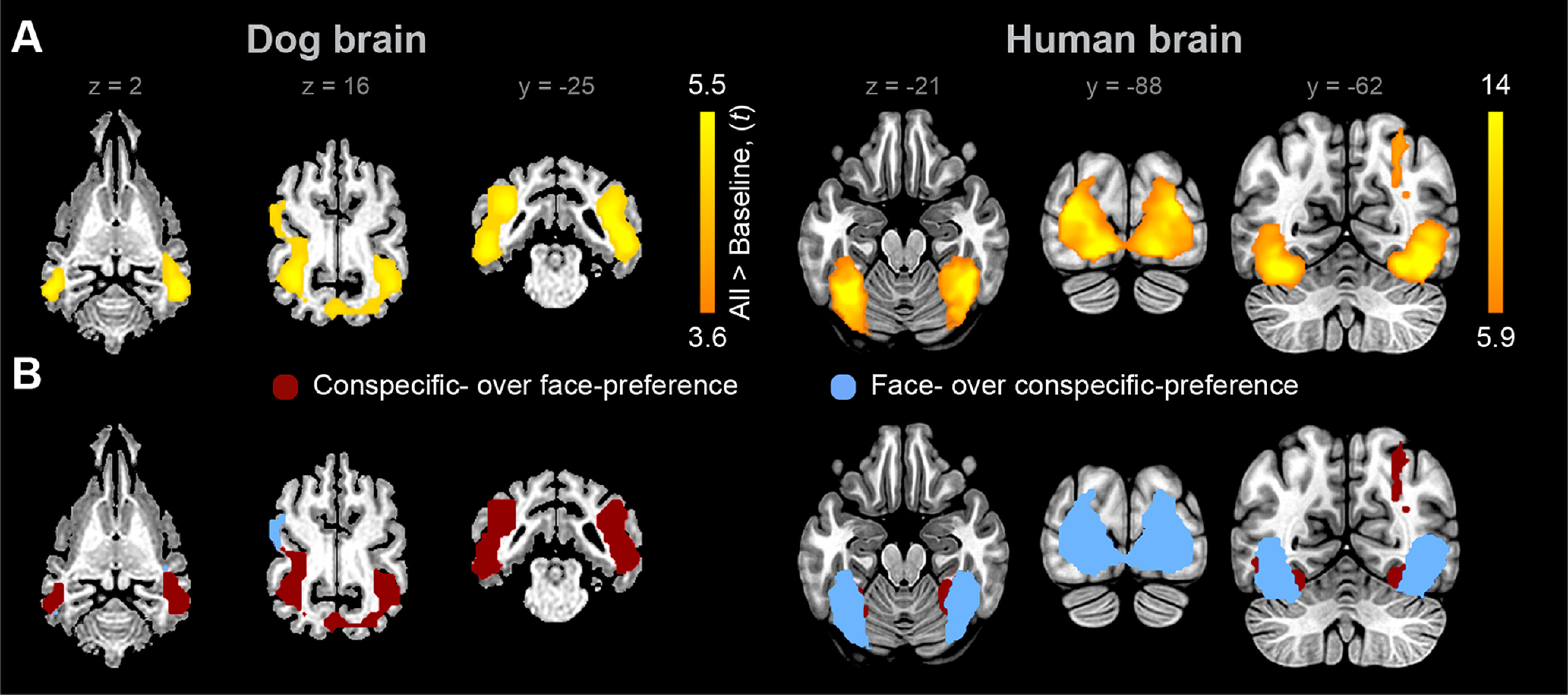
Visually-responsive regions and processing preference differences in dogs and humans. ***A***, Visually-responsive regions (color coded with warm) as determined by the contrast of experimental conditions versus fixation baseline in the dog brain (left), thresholded at *p* < 0.001 uncorrected and *p* < 0.05 cluster-corrected for FWE and in the human brain (right), thresholded at *p* < 0.000001 uncorrected and *p* < 0.001 cluster-corrected for FWE. ***B***, Group-level binary map of stronger conspecific-preference than face-preference (red) and stronger face-preference than conspecific-preference (blue) in visually-responsive regions. See Results for corresponding permutation statistics comparing the proportions of voxels with either preference and on random effects analyses of individual binary preference maps. See also Extended Data Figures 2-1, 2-2.

Analyses of the extent to which visually-responsive voxels respond stronger to the conspecificity or to the faceness of stimuli indicated that in dogs, 94.6% of the visually-responsive cortex showed greater preference for conspecificity than for faces (likelihood of obtaining the observed proportions by chance, using permutation testing: *p* < 0.01). In humans, 10.8% of the visually-responsive cortex showed this pattern (*p* < 0.05). Consequently, 5.4% of dog and 89.2% of human visually-responsive cortex showed greater preference for faces than for conspecificity (Fig. 2).

10.1523/JNEUROSCI.2800-19.2020.f2-1 Figure 2-1 Visually-responsive regions in the dog and human brain (all conditions vs. baseline). Download Figure 2-1, DOCX file

10.1523/JNEUROSCI.2800-19.2020.f2-2 Figure 2-2 Results of non-parametric random effects analyses of individual binary preference maps within visually-responsive regions in the dog and human brain. Download Figure 2-2, DOCX file

Non-parametric group analyses of the subject-level binary response preference maps (Extended Data Fig. 2-2) showed that, in dogs, the bilateral mSSG and a splenial gyrus (SpG) cluster exhibited greater conspecific-preference than face-preference, and these clusters were overlapping with those responding stronger to dog relative to human stimuli. In humans, the opposite pattern emerged: a bilateral IOG cluster and a right inferior temporal gyrus (ITG) cluster exhibited greater face-preference than conspecific-preference, and these clusters were overlapping with those responding stronger to face than to occiput stimuli.

### MVPA

**Figure 3.**
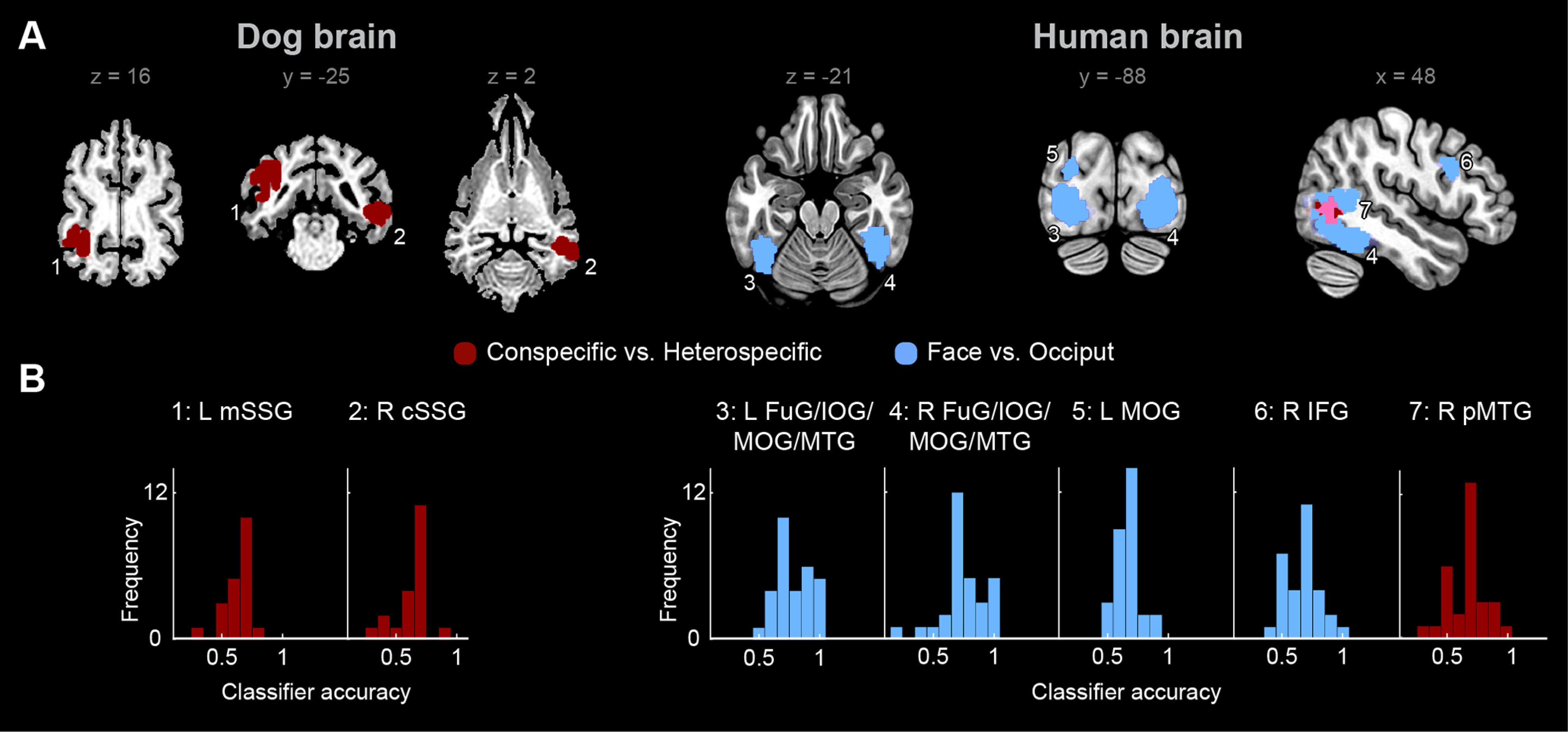
MVPA using searchlight. ***A***, Brain regions within the visually-responsive cortex of dogs and humans that discriminate conspecific from heterospecific (red) and face from occiput (blue) stimuli. The mean classifier accuracy significance level (*p*) on each voxel was calculated using permutation testing (see Materials and Methods) *p* < 0.001 uncorrected and *p* < 0.05 cluster-corrected for FWE for dogs and *p* < 0.000001 uncorrected and *p* < 0.001 cluster corrected for FWE for humans, the searchlight used a spherical kernel with a radius of 4 mm for dogs and 8 mm for humans. ***B***, Histograms depicting classification accuracy across participants for each cluster peak. L = left; R = right; cSSG = caudal ectosylvian gyrus; mSSG = mid suprasylvian gyrus; FuG = fusiform gyrus; IFG = inferior frontal gyrus; IOG = inferior occipital gyrus; ITG = inferior temporal gyrus; MOG = middle occipital gyrus; pMTG = posterior middle temporal gyrus. See also Extended Data Figure 3-1.

We found two clusters in dogs for the C versus He comparison, one in the left mSSG, with group mean classifier accuracy M = 0.642, SD = 0.124 and one in the right caudal suprasylvian gyrus (cSSG), M = 0.629, SD = 0.136. No clusters were revealed in dogs for the F versus O comparison. In humans, a cluster was revealed for the C versus He comparison, in the right pMTG, M = 0.675, SD = 0.163. Four clusters were revealed for the F versus O comparison: a large cluster including parts of the right FuG, IOG, MOG, and MTG, M = 0.761, SD = 0.180, a large cluster including parts of the left FuG, IOG, MOG, and MTG, M = 0.797, SD = 0.148, the right inferior frontal gyrus (IFG), M = 0.672, SD = 0.152, and a left MOG cluster, M = 0.667, SD = 0.112. All results were cluster corrected for FWE *p* < 0.05 for dogs and *p* < 0.001 for humans (Fig. 3; for the full list of peaks and subpeaks, see Extended Data Fig. 3-1).

10.1523/JNEUROSCI.2800-19.2020.f3-1 Figure 3-1 Results from MVPA within visually-responsive regions in the dog and human brain. Download Figure 3-1, DOCX file

### RSA

**Figure 4.**
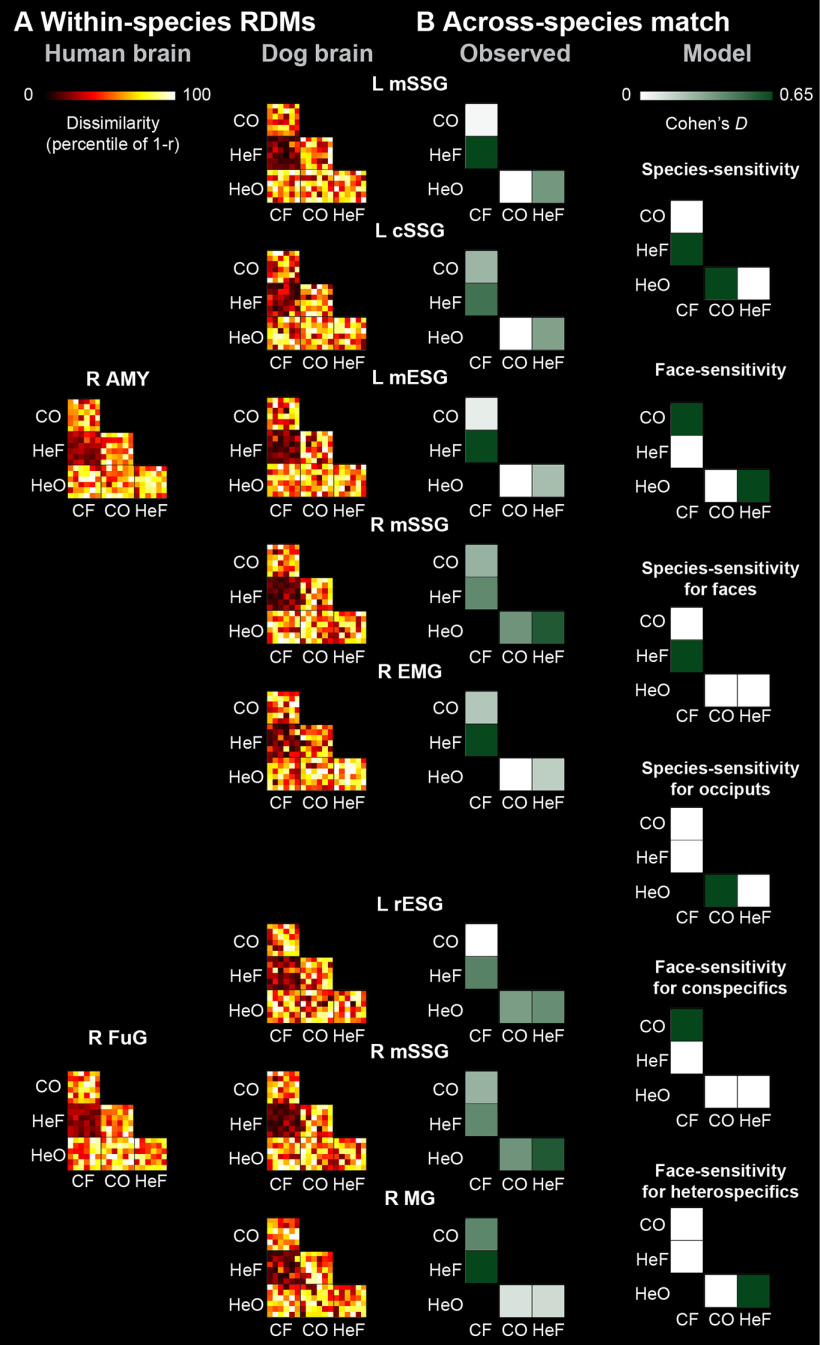
Across-species RSAs. ***A***, RDMs between select GLM-derived human peaks (first column, sphere radius = 8 mm) and matching dog brain peaks (second column, sphere radius = 4 mm) using a searchlight approach (one sample *t* test, *p* < 0.001 uncorrected and *p* < 0.05 cluster corrected for FWE), in visually-responsive regions. All RDMs are represented as percentile of Pearson distance (1 – Pearson correlation). ***B***, Observed effect sizes (Cohen's *d*) for the across-species matching of RDMs for each peak-pair (first column), and modelled effect size patterns reflecting potential driving forces underlying across-species matching (second column); see also Extended Data Figure 4-3. C = conspecific; He = heterospecific; F = face; O = occiput; L = left; R = right; AMY = amygdala/hippocampus; FuG = fusiform gyrus; cSSG = caudal suprasylvian gyrus; EMG = ectomarginal gyrus; mESG = mid ectosylvian gyrus; MG = marginal gyrus; mSSG = mid suprasylvian gyrus; rESG = rostral ectosylvian gyrus. See also Extended Data Figures 4-1, 4-2, 4-3.

Across-species RSA using the direct matching model indicated no visually-responsive dog regions that represented stimuli similarly to the GLM-derived human regions. Across-species RSA using the functional matching model showed that the canine left mid ectosylvian gyrus (mESG), *t*_(29)_ = 4.994, right ectomarginal gyrus (EMG), *t*_(29)_ = 4.882, left cSSG, *t*_(29)_ = 4.732 and right and left mSSG, *t*_(29)_ = [6.378 and 4.997] represented stimuli similarly to the human right AMY (*p*s < 0.001), and the canine left rESG, *t*_(29)_ = 4.383, right MG, *t*_(29)_ = 4.741 and right mSSG, *t*_(29)_ = 4.632 represented stimuli similarly to the human right FuG (*p*s < 0.001; Fig. 4). Follow-up pairwise comparisons indicated that a medium species effect for faces (i.e., HeF-CF) drove the representational similarity effect between the dog left (*d* = 0.657) and right mSSG (*d* = 0.581), left mESG (*d* = 0.640), and right EMG (*d* = 0.641), and the human right AMY; a medium species effect for faces in case of the representational similarity between the dog right MG (*d* = 0.656) and the human right FuG; and a medium faceness effect for heterospecifics (i.e., HeF-HeO) in case of the representational similarity between the dog right mSSG (*d* = 0.580) and the human right FuG. All across-species RSA results are summarized in Extended Data Figures 4-1, 4-2, 4-3.

10.1523/JNEUROSCI.2800-19.2020.f4-1 Figure 4-1 RSA results on dog brain regions with similar activity pattern as select peaks from the human brain. Download Figure 4-1, DOCX file

10.1523/JNEUROSCI.2800-19.2020.f4-2 Figure 4-2 RSA results on dog visually-responsive brain regions with similar activity pattern as select peaks from the human brain. Download Figure 4-2, DOCX file

10.1523/JNEUROSCI.2800-19.2020.f4-3 Figure 4-3 Follow-up analyses on effect sizes corresponding to RSA effects observed between visually-responsive dog brain regions and peaks of select human brain regions. Download Figure 4-1, DOCX file

### Individual difference-focused analyses

To determine whether lack of support for face sensitivity in dogs generalizes across the 20 dogs tested, we assessed for face-preference in each dog, by testing whether there is suprathreshold F > O or DF>DO sensitivity in any individual. Using a *p* < 0.001 uncorrected voxel threshold on individual contrast maps, we found that no dogs had a meaningful number of suprathreshold face-preferring voxels (three dogs had such F > O voxels, M_nr of voxels_ = 1.33, range 1–2; two dogs had such DF>DO voxels, M_nr of voxels_ = 2.5, range 2–3). In comparison, similarly thresholded individual D > H contrast maps yielded sizeable clusters in many dogs (10 dogs had such voxels, M_nr of voxels_ = 61, range 1–227).

To assess for any effects that relevant dog individual difference variables may have had on our results, experience and breeding variables (for details, see Extended Data Table 1-4) were entered into GLM analyses as covariates to assess their effects on HF-preference (quantified in the HF>HO and HF>DF contrasts) in the visually-responsive cortex of dogs. To index “experience,” the type of training each dog received was considered, quantifying the degree to which such training was face-oriented (involved/necessitated attending to human faces) on a four-point scale. To index “breeding,” a brain-based cephalic index was calculated for each dog. Not only is a brain-based cephalic index appropriate to quantify the effects of breeding on the architecture of the dog brain (Hecht et al., 2019), it is also relevant with regard to attraction to human faces in dogs (Bognár et al., 2018). Findings indicated neither individual difference variable covaried with HF-preference, neither at a more standard (*p* < 0.001), nor at a more liberal voxel threshold (*p* < 0.01), *p* < 0.05 cluster-corrected for FWE.

To assess for any effects that relevant human individual difference variables may have had on our results, self-reported dog ownership (as a proxy for expertise), was entered into GLM analyses as a covariate. We assessed the covariate effect on D > H, DF>HF and DF>DO responses, interest was in whether individuals who owned a dog would show greater responses to dog stimuli overall, or to dog face stimuli specifically, compared with those who did not own a dog, in the visually responsive cortex of humans. Results indicated that expertise covaried with D > H response in the right lingual gyrus (LiG; an 11-voxel-large cluster, peak at 8, −80, −8; thresholded at *p* < 0.000001 uncorrected and *p* < 0.001 cluster-corrected for FWE). This pattern was driven by a difference in dog owners (*n* = 11), who showed greater right LiG response to dog (M = 3.212, SD = 1.628) than human stimuli (M = 3.212, SD = 1.628), *t*_(10)_ = 6.934, *p* < 0.001. In non-owners (*n* = 19), R LiG response was not affected by species, *t*_(18)_ = 1.459, *p* = 0.162. Expertise did not covary with DF>HF or DF>DO response.

## Discussion

Univariate and MVPAs identified species-sensitive visual regions in both human and dog brains, but face-sensitive regions in humans only. Our findings also demonstrate that the relative roles of conspecific-preference and face-preference in visuo-social perception differ between humans and dogs. In humans, all conspecific-preferring regions were face-preferring, whereas in dogs, none of the conspecific-preferring regions exhibited face-preference. Direct comparisons of conspecific-preference and face-preference in the visually-responsive cortex confirmed this difference in the relative roles of processing preferences across species. In humans, only regions exhibiting greater face-preference than conspecific-preference were identified. In contrast, in dogs, only regions exhibiting greater conspecific-preference than face-preference were identified. These results imply that, unlike in humans, face-preference is not primary to conspecific-preference in the dog visually-responsive cortex.

### Face-preference

Regarding face-preference, in humans, the cortical regions that showed stronger response to faces relative to occiputs corresponded to key structures of the face network (Duchaine and Yovel, 2015). In contrast, in dogs, no cortical regions preferred faces to occiputs. Accordingly, although neural face sensitivity appears general across primates, it may not be a general organizing principle of visuo-social perception across mammals. Neural face sensitivity does not appear to be such an organizing principle in dogs, who, e.g., for assessment of attentional or motivational state, rely less on information in faces and more on information in larger bodily units (Emery, 2000). Related, in dogs, there is no evidence that for kin recognition or mate selection facial cues would be more important than non-facial bodily cues, acoustic or chemical signals (Leopold and Rhodes, 2010). However, behaviorally, dogs are attracted to faces (Gácsi et al., 2004; Adachi et al., 2007) and can differentiate dog from human faces (Racca et al., 2010), though this ability is limited: even after training, only a minority (20%) can discriminate their owner's and a stranger's face in the absence of head-contour (but with eyes, mouth, and nose clearly visible; Huber et al., 2013). All current and prior data considered, we propose that our results are reconcilable with earlier neuroimaging findings that indicated face-preferring dog brain regions based on faces versus objects (Dilks et al., 2015; Cuaya et al., 2016) and human faces versus dog faces (Dilks et al., 2015; Thompkins et al., 2018) comparisons. As further support for reconcilability of current and these past findings, none of the earlier studies involved examination of face-preference, controlling for animate-inanimate and conspecific-heterospecific confounds. Of note, consistent with the current results, no face-preference was observed in earlier studies to faces versus scrambled faces comparisons (Dilks et al., 2015; Szabó et al., 2020). In these prior studies, however, pertinent comparisons were not of dog faces versus scrambled dog faces (Dilks et al., 2015 report data for dog and human faces pooled together and Szabó et al., 2020 for human faces only). Accordingly, although the corresponding findings may be indicative of lack of face-preference in dogs, those may also reflect limitations of chosen experimental stimuli. Contrasts involving conspecific stimuli, rather than human stimuli, may be more sensitive to probe face sensitivity in dogs. Nevertheless, in further support of our conclusion, we observed neither any clusters with greater response to DF>DO (Extended Data Table 1-1), nor a meaningful number of suprathreshold face-preferring (F > O or DF>DO) voxels in any individual dog.

It is important to note that our negative findings are not conclusive evidence against dog face areas. It is possible that our measurement settings may have not been sufficiently sensitive. However, the (1) relatively high number of dogs tested (compared with prior neuroimaging studies), (2) consistency between the herein and earlier identified (Dilks et al., 2015) dog visually-responsive areas, (3) clear positive effects for the D versus H contrast in dogs, (4) clear F versus O effects for the same stimuli in humans, and (5) consistency of our univariate (macromap-level) and MVPA (micromap-level; Dehaene and Cohen, 2007) findings, in combination, make the measurement insensitivity explanation unlikely. Instead, across-study differences in findings of face-preference may reflect differences in control conditions, underscoring the importance of re-assessing earlier claims of dog face areas using stricter controls. It is further possible that the lack of observed face-preferring regions in dogs can be partly explained by power issues, i.e., it may have been a result of our “strict” threshold that we did not detect a weak face-preference effect in our (lower-than-human quality) dog data. However, that we found strong conspecific effects in dogs suggests otherwise. Also, that at the group level, even a lower threshold did not indicate a face-preference effect, and at the individual level, no dogs had a meaningful number of face-preferring voxels make this improbable.

### Conspecific-preference

Findings of conspecific-preferring regions in the visually-responsive cortex of humans and dogs support the hypothesis that, similarly to the auditory modality (Petkov et al., 2008; Andics et al., 2014), neural conspecific-preference is present in phylogenetically distant mammal species in the visual modality. In dogs, we identified a robust conspecific-preferring cluster in the bilateral mSSG; a visual association area at the parieto-temporo-occipital junction (Kowalska, 2000). The involvement of the mSSG in visuo-social perception is consistent with corresponding regions having been broadly implicated in visual processing in cats (Dow and Dubner, 1971; Yin and Greenwood, 1992) and marmosets (Hupfeld et al., 2007), with homologies across the cat suprasylvian sulcus and the macaque V5 (involved in early visual processing; Payne, 1993) and the cat mSSG and monkey inferior parietal lobe (IPL; involved in directing visual attention; Krüger et al., 1993). In humans, only face-preferring regions [specifically, the pMTG, the aMTG (for faces) and the AMY] showed conspecific-preference. This corroborates previous findings of the AMY being conspecific-preferring (Blonder et al., 2004). Within the face network, both AMY and pMTG are thought to be involved in emotional cue processing (Duchaine and Yovel, 2015), our findings may thus reflect a greater relevance of conspecificity in emotional than in structural information processing for faces in humans. Regarding the right aMTG, our findings are consistent with earlier results indicating this region is involved in dynamic human face processing (Duchaine and Yovel, 2015) and suggest that, similarly to ventral subregions of the face-sensitive anterior temporal lobe (Collins and Olson, 2014), this dorsal face area prefers conspecific face stimuli.

Conspecific-preference, as observed here in the dog parieto-temporo-occipital junction, a region purportedly involved in structural processing, may be of a different nature than face-preference, as observed in the human occipito-temporal cortex. The hypothesized underlying neural mechanism behind face-preference in the human visual cortex is category selectivity (Kanwisher, 2017; Op de Beeck et al., 2019). Conspecific-preference, however, may also be explainable by sensitivity to motivational relevance, a mechanism that in humans modulates visual cognition through attention (Summerfield and Egner, 2009), and not category selectivity. In support, in humans, we observed conspecific-preference only in (face-preferring) regions involved in emotional cue processing (Duchaine and Yovel, 2015) but not in (face-preferring) regions involved in structural processing. Additionally, fine-grained, feature-based category selectivity in visual processing may be better developed in species with greater visual acuity, such as primates (Leopold and Rhodes, 2010), but less so in species with poorer visual acuity, such as dogs (Odom et al., 1983; Pongrácz et al., 2017). In the absence of empirical data, it remains an open question whether conspecific-preference is driven by category selectivity or motivational relevance in the dog visual cortex.

### Neural mechanisms controlling processing preferences

Processing preferences for natural stimulus classes may not necessarily reflect functional distinctions. Rather, such differences may be explained by sensitivity to visual similarity (Kriegeskorte et al., 2008a). In our findings, differences in processing preferences being driven by functional distinctions are supported by results of two analyses. First, all species and face main effects were unchanged when controlling for differences in low-level visual properties across conditions. Second, it was only in the functional matching RSA model (i.e., when representation of dog stimuli in dogs was matched with representation of human stimuli in humans and vice versa), but not in the direct matching RSA model (i.e., when representation of dog stimuli in dogs was matched with representation of dog stimuli in humans and vice versa) that we identified dog regions with a response pattern comparable to any human face-preferring or conspecific-preferring region's response pattern. Specifically, visually-responsive dog regions, involving the mSSG, showed representational similarity to the human FuG and AMY in the functional matching model. Arguably, this functional matching model advantage indicates that response pattern similarities reference a relative, motivationally relevant distinction between conspecific and heterospecific stimuli to the perceiver, rather than absolute visual differences between dog and human stimuli. Of note, representational similarities across species were primarily driven by species distinctions for faces. Accordingly, visual conspecific-preference for faces may involve functionally analog neural response patterns in dogs and humans.

### Effects of individual differences in dogs and humans

In dogs, we found no evidence to indicate that individual differences in experience with human faces or breeding-related structural properties systematically affect brain response to human faces. Of note, our sample was relatively homogeneous in these aspects; all 20 dogs were highly trained (similar to Dilks et al., 2015) family dogs, regularly exposed to human faces (as such, any experience-related bias in this sample would have been in the direction of increased likelihood of human face sensitivity). Further, most dogs represented modern, cooperative breed types. Thus, although generalizing our findings across all domestic dogs in absence of a more heterogeneous sample may be inappropriate, there is no reason to assume that dogs with less experience or dogs representing basal or non-cooperative breed types would show greater neural human face sensitivity. Finally, although brain shape varied across the sample, all dogs were mesocephalic (medium-headed). Given a potential association between differences in cephalic index (Hecht et al., 2019) and readiness to attend to faces (Bognár et al., 2018), additional research with brachycephalic (short-headed) dogs may be informative.

In humans, regarding individual differences in experience, findings are both consistent with and extend prior findings, in indicating that participants who owned a dog, unlike those who did not, exhibited greater right LiG response to dog than to human stimuli. It has been argued that real-world expertise shapes human behavior and neural processing (Harel et al., 2013). Neural evidence suggests that experts exhibit greater brain response to objects of expertise than to other objects throughout (and outside of) the visual cortex (Harel et al., 2013), including the FFA (Gauthier et al., 2000; Xu, 2005), collateral sulcus/LiG, precuneus, and STS (Harel et al., 2010; McGugin et al., 2012). Dog ownership can be conceptualized as real-world expertise. Relevant behavioral evidence indicates that dog experts (i.e., dog show judges) have enhanced recognition of individual dogs (only) of the specific breeds with which they are familiar (Diamond and Carey, 1986; Robbins and McKone, 2007). We suggest that the activity pattern we found in the right LiG is thus consistent with an account of expertise-based individual differences in human visual processing. Notably, we found no such expertise effects in any other brain regions.

### Potential mechanisms for greater response to heterospecific and occiput stimuli in humans

In humans, greater response to heterospecific than conspecific stimuli was observed in the (also face-preferring) IOG/LOC; left SOG; and in bilateral LOTC. Finally, in a large bilateral cluster including parts of FuG, IOG, MOG and SOG, response was weaker to human than to dog faces (or human occiputs). Greater response to occiput than face stimuli was also observed mainly in regions associated with visual functions, i.e., the left MOG, the PCUN, the left IPL and the right mFuG; and also in the right SFG. There are a handful of accounts, albeit related, presuming different mechanisms, that may explain observed greater response to heterospecific and occiput stimuli. Which, if any of these accounts best explains these results, cannot be determined in the absence of further control conditions and the current study was not designed to do so.

First, increased processing demands (e.g., because of addition of phase noise to face stimuli) are associated with greater bilateral LOC (Bankó et al., 2011) and bilateral MOG (Hermann et al., 2015) response and processing heterospecific and occiput stimuli may be more effortful. Second, norm-based processing involves evaluation of degree to which a stimulus differs from a prototype (Rhodes et al., 2005). Face stimuli further from the prototype generate stronger neural responses in face-sensitive brain regions in humans (Loffler et al., 2005; Tsao and Livingstone, 2008) and monkeys (Leopold et al., 2006). Conspecific (face) stimuli may better match a potentially referenced (face) prototype. Third, findings may be explainable by a novelty effect; others found greater response to novel relative to familiar stimuli in the IOG (Kiehl et al., 2001; Ousdal et al., 2014; Geiger et al., 2018; Manahova et al., 2018) and heterospecific and occiput stimuli are arguably less familiar than conspecific and face stimuli. Fourth, others observed greater response in the SOG to dog barking/monkey lipsmacking than human lipreading (Buccino et al., 2004) and the LOTC to human bodies/body parts than human faces (Lingnau and Downing, 2015). Representations of the human body may extend to animals (Konkle and Caramazza, 2013), although such animal/body category-sensitive regions are small.

### Lateralization

Regarding lateralization, human temporal and limbic structures implicated here showed greater involvement of the right hemisphere. In both the pMTG and the AMY, both conspecific-preference and face-preference were observed only on the right side. In the pMTG, direct hemispheric comparisons confirmed a right bias in face-preference. In the aMTG, face-preference was observed only in the right hemisphere. These findings of right hemispheric dominance are consistent with prior behavioral and neural studies on face perception (Duchaine and Yovel, 2015). Of note, the human ventral face-selective areas exhibited no clear right-hemisphere dominance of face-preference in the present study. This may be explained by our use of occiputs as comparison stimuli. Although traditionally reported core- and extended face network regions were identified by our face versus occiput contrast, a different response pattern from that for e.g., faces versus objects (as was done in studies indicating lateralization in the human FFA; Kanwisher et al., 1997; Kanwisher and Yovel, 2006) may have been elicited by it. This finding may also be explained by our relatively more coarse and macro-level design, experimental manipulations, and peak selection (Rossion, 2014). Finally, visual association areas revealed by our contrasts in dogs exhibited no lateralization in conspecific-preference. This is consistent with earlier findings on human and dog auditory conspecificity processing in auditory association areas (Andics et al., 2014).

## Summary

The research presented here constitutes the first directly comparative, noninvasive visual neuroimaging study of a non-primate and a primate species. We presented neuroimaging evidence for visual species sensitivity in both dogs and humans and showed that in dogs, conspecific-preference is primary over face-preference whereas in humans, face-preference is primary over conspecific-preference. Further, we identified dog and human brain regions with a similar representational pattern for processing visuo-social stimuli, and this similarity effect was mainly driven by species distinctions based on faces. Together, these results indicate functional analogies in dog and human visuo-social processing of conspecificity but suggest that cortical specialization for face perception may not be ubiquitous across mammals.
