# Supplementary material for: Comparative Brain Imaging Reveals Analogous and Divergent Patterns of Species and Face Sensitivity in Humans and Dogs
Source: J Neurosci. 2020 Oct 21;40(43):8396–408. doi: 10.1523/JNEUROSCI.2800-19.2020 (PMC7577605; doi:10.1523/JNEUROSCI.2800-19.2020)
Supplement: Table 1-1 — Extended GLM results for dogs and humans. Download Table 1-1, DOCX file [file ns-JN-RM-2800-19-s02.docx]

Table 1–1

*Extended GLM results for dogs and humans.*

| Contrast Brain region | | Cluster *p*  (FWE-corr) Dogs | Cluster size  (voxels) | Peak  T | Coordinates  (x, y, z) |
| --- | --- | --- | --- | --- | --- |
| HF>HO | - | - | - | - | - |
| HO>HF | - | - | - | - | - |
| HF>DF | - | - | - | - | - |
| DF>HF | L mSSG | .004 | 52 | 5.646 | -14, -28, 20 |
|  | R cSSG | .017 | 36 | 4.864 | 22, -26, 2 |
| HF>DO | - | - | - | - | - |
| DO>HF | - | - | - | - | - |
| HO>DF | - | - | - | - | - |
| DF>HO | R mSSG | <.001 | 187 | 8.140 | 18, -28, 20 |
|  | L MG |  |  | 7.178 | 0, -36, 22 |
|  | R cSSG |  |  | 4.892 | 20, -28, 0 |
|  | L mSSG | .011 | 40 | 4.686 | -14, -30, 20 |
| HO>DO | - | - | - | - | - |
| DO>HO | R mSSG | .001 | 76 | 5.289 | 18, -30, 18 |
| DF>DO | - | - | - | - | - |
| DO>DF | - | - | - | - | - |
|  |  | Humans |  |  |  |
| HF>HO | R MTG | <.001 | 827 | 10.421 | 52, -50, 10 |
|  |  |  |  | 8.835 | 50, -12, -14 |
|  |  |  |  | 8.191 | 56, -30, -4 |
|  |  |  |  | 6.931 | 50, 4, -26 |
|  | R HPC | <.001 | 88 | 8.658 | 22, -8, -12 |
|  | L MTG | <.001 | 51 | 7.519 | -50, -46, 12 |
|  | R ITG | .001 | 30 | 7.077 | 46, -48, -24 |
| HO>HF | L LiG | <.001 | 342 | 8.033 | -24, -60, -12 |
|  | R CUN | <.001 | 441 | 7.898 | 6, -82, 42 |
|  | L PCUN |  |  | 6.530 | -10, -68, 56 |
|  | R PCUN |  |  | 6.195 | 6, -74, 56 |
|  | R FuG | <.001 | 547 | 7.892 | 24, -58, -10 |
|  |  |  |  | 7.192 | 22, -42, -16 |
|  | L MOG | <.001 | 164 | 7.331 | -40, -80, 16 |
|  |  |  |  | 7.078 | -24, -82, 20 |
|  | L SOG | <.001 | 114 | 7.310 | -26, -76, 42 |
|  | R SOG | <.001 | 222 | 7.153 | 28, -80, 22 |
|  | L IPG | <.001 | 40 | 7.064 | -56, -30, 42 |
|  | L ITG | <.001 | 42 | 6.930 | -52, -62, -10 |
|  | L SFGdor | <.001 | 36 | 6.348 | -20, 6, 60 |
| HF>DF | R MTG | <.001 | 423 | 8.530 | 52, -50, 10 |
|  |  | <.001 | 93 | 8.091 | 50, -4, -22 |

|  | R HPC | <.001 | 57 | 7.450 | 20, -10, -16 |
| --- | --- | --- | --- | --- | --- |
| DF>HF | R SOG | <.001 | 4520 | 11.172 | 24, -84, 20 |
|  | L IOG |  |  | 10.417 | -42, -78, 2 |
|  | R FuG |  |  | 9.464 | 22, -72, -14 |
|  | R CUN |  |  | 9.434 | 8, -92, 16 |
|  | L ITG |  |  | 9.093 | -46, -64, -8 |
|  | R ITG |  |  | 8.782 | 48, -68, -8 |
|  | L LiG |  |  | 8.610 | -20, -80, -14 |
|  | L MOG |  |  | 8.569 | -20, -86, 16 |
| HF>DO | R MTG | <.001 | 526 | 9.610 | 52, -50, 10 |
|  |  |  |  | 7.013 | 56, -30, -2 |
|  | R HPC | <.001 | 238 | 9.385 | 20, -6, -14 |
|  | R MTG | <.001 | 182 | 9.054 | 50, -6, -18 |
|  | R IOG | <.001 | 155 | 9.008 | 28, -94, -4 |
|  | R LiG | <.001 | 36 | 8.011 | 4, -30, -4 |
|  | R PreCG | <.001 | 60 | 7.579 | 42, 4, 40 |
|  | L IOG | <.001 | 54 | 7.472 | -28, -92, -6 |
|  | L HPC | <.001 | 91 | 7.287 | -18, -6, -14 |
|  | R ITG | <.001 | 33 | 6.779 | 44, -50, -24 |
| DO>HF | R ITG | <.001 | 168 | 7.993 | 52, -62, -8 |
|  | L IPG | <.001 | 94 | 7.744 | -52, -30, 42 |
|  | L PCUN | <.001 | 604 | 7.701 | -6, -76, 54 |
|  | R CUN |  |  | 7.274 | 12, -82, 40 |
|  | L SOG |  |  | 6.940 | -22, -86, 30 |
|  | L CUN |  |  | 6.372 | -6, -76, 32 |
|  | L MTG | <.001 | 198 | 7.533 | -52, -66, 0 |
|  | R CN | <.001 | 34 | 7.479 | 6, 22, 10 |
|  | L MOG | .001 | 30 | 7.374 | -26, -78, 40 |
|  | R SPG | <.001 | 39 | 7.032 | 30, -52, 66 |
| HO>DF | R PCUN | <.001 | 51 | 7.901 | 14, -54, 58 |
| DF>HO | L IOG | <.001 | 538 | 12.688 | -40, -82, -8 |
|  | R IOG | <.001 | 540 | 9.933 | 30, -88, -8 |
|  |  |  |  | 8.022 | 42, -74, -10 |
|  | L FuG | <.001 | 179 | 9.910 | -40, -52, -22 |
|  | R FuG | <.001 | 76 | 7.495 | 44, -44, -22 |
|  | R CUN | <.001 | 42 | 7.229 | 12, -96, 10 |
|  | L SOG | <.001 | 100 | 7.174 | -8, -96, 8 |
| HO>DO | R IOG | <.001 | 32 | 6.701 | 28, -94, -4 |
|  | L MOG | <.001 | 40 | 6.613 | -24, -94, -2 |
| DO>HO | R MTG | <.001 | 31 | 7.015 | 44, -60, 4 |
| DF>DO | R IOG | <.001 | 1727 | 15.165 | 28, -88, -2 |
|  | R FuG |  |  | 12.583 | 40, -46, -20 |
|  |  |  |  | 11.065 | 40, -66, -16 |

|  | L FuG |  |  | 11.014 | -38, -54, -20 |
| --- | --- | --- | --- | --- | --- |
|  |  |  |  | 10.271 | -38, -72, -18 |
|  | L CAL |  |  | 7.431 | -6, -96, 6 |
| DO>DF | R PCUN | <.001 | 39 | 7.355 | 30, -52, 0 |

*Note.* Threshold for reporting for all contrasts was *p*<.000001 and cluster *p*<.001 for humans and *p*<.001 and

| R CAL | 8.380 | 14, -98, 6 |
| --- | --- | --- |
| L IOG <.001 1982 12.720 -30, -88, -4 | | |

cluster *p*<.05 for dogs. L=left; R=right; mSSG=mid suprasylvian gyrus; cSSG=caudal suprasylvian gyrus; MG=marginal gyrus; MTG=middle temporal gyrus; HPC=hippocampus; ITG=inferior temporal gyrus; LiG=lingual gyrus; CUN=cuneus; PCUN=precuneus; FuG=fusiform gyrus; MOG=middle occipital gyrus; SOG=superior occipital gyrus; IPG=inferior parietal gyrus, excluding supramarginal and angular gyri; ITG=inferior temporal gyrus; SFGdor= dorsolateral superior frontal gyrus; IOG=inferior occipital gyrus;

PreCG=precentral gyrus; CN=caudate nucleus; SPG= superior parietal gyrus; CAL=calcarine sulcus.
