# Supplementary material for: Comparative Brain Imaging Reveals Analogous and Divergent Patterns of Species and Face Sensitivity in Humans and Dogs
Source: J Neurosci. 2020 Oct 21;40(43):8396–408. doi: 10.1523/JNEUROSCI.2800-19.2020 (PMC7577605; doi:10.1523/JNEUROSCI.2800-19.2020)
Supplement: Table 1-2 — Parametric modulation effects of basic visual properties (uncorrected). Download Table 1-2, DOCX file [file ns-JN-RM-2800-19-s03.docx]

Table 1–2

*Parametric modulation effects of basic visual properties (uncorrected).*

|  | *t* | *df* | *p* |
| --- | --- | --- | --- |
| R IOG - brightness | -3.588 | 29 | .001 |
| R pMTG - contrast | 3.453 | 29 | .001 |
| R pMTG - brightness | 3.301 | 29 | .002 |
| L pMTG - saturation | 2.148 | 29 | .040 |

*Note.* L=left; R=right; IOG=inferior occipital gyrus; pMTG=posterior middle temporal

gyrus. 18 comparisons were n.s., *p*s>.05 uncorr.

6
