# Supplementary material for: Comparative Brain Imaging Reveals Analogous and Divergent Patterns of Species and Face Sensitivity in Humans and Dogs
Source: J Neurosci. 2020 Oct 21;40(43):8396–408. doi: 10.1523/JNEUROSCI.2800-19.2020 (PMC7577605; doi:10.1523/JNEUROSCI.2800-19.2020)
Supplement: Table 1-3 — ANOVA face and species main effects with all stimuli and with visually most deviant stimulus blocks excluded. Download Table 1-3, DOCX file [file ns-JN-RM-2800-19-s04.docx]

Table 1–3

*ANOVA face and species main effects with all stimuli and with visually most deviant stimulus blocks excluded.*

All stimuli Visually controlled stimuli

Main effect *F p F p*

Dogs mSSG

| face | .038 | .846 | .002 | .963 |
| --- | --- | --- | --- | --- |
| species | 23.494 | <.001 | 10.219 | .002 |

Humans

IOG

| face | 49.538 | <.001 | 54.668 |  | <.001 | |  |
| --- | --- | --- | --- | --- | --- | --- | --- |
| species | .365 | .546 | .313 |  | .576 | |  |
|  |  | FuG |  |  |  | |  |
| face | 36.391 | <.001 | 38.328 |  | <.001 | |  |
| species | .085 | .771 | .288 |  | .592 | |  |
|  |  | pMTG |  |  |  | |  |
| face | 28.637 | <.001 | 30.467 |  | <.001 | |  |
| species | 5.344 | .022 | 3.410 |  | .066 | |  |
|  |  | AMY |  |  |  | |  |
| face | 29.563 | <.001 | 22.072 |  | <.001 | |  |
| species | 20.205 | <.001 | 21.400 |  | <.001 | |  |
|  |  | aMTG |  |  |  | |  |
| face | 27.595 | <.001 | 24.822 |  | | <.001 | |
| species | 6.462 | .012 | 6.398 |  | | .013 | |

*Note.* mSSG=mid suprasylvian gyrus; IOG=inferior occipital gyrus; FuG=fusiform gyrus; pMTG=posterior middle temporal gyrus; AMY=amygdala/hippocampus; aMTG=anterior middle temporal gyrus;.

7
