# Supplementary material for: Comparative Brain Imaging Reveals Analogous and Divergent Patterns of Species and Face Sensitivity in Humans and Dogs
Source: J Neurosci. 2020 Oct 21;40(43):8396–408. doi: 10.1523/JNEUROSCI.2800-19.2020 (PMC7577605; doi:10.1523/JNEUROSCI.2800-19.2020)
Supplement: Table 1-4 — Individual differences with regard to breed, human face-related experience, and cephalic index across dogs. Download Table 1-4, DOCX file [file ns-JN-RM-2800-19-s05.docx]

Table 1–4

*Individual differences with regard to breed, human face-related experience, and cephalic index across dogs.*

Brain-based

ID

cephalic index Breed Type of training

Face-orientation of training

DHDH001 70.476 golden retriever therapy advanced, agility 3

DHDH002 77.899 mix Mantrailing, schutzhund (defense) 1

DHDH003 69.065 golden retriever therapy basic, K99/basic 2 (obedience, distance work, tricks)

DHDH004 70.000 golden retriever assistance, agility 4

DHDH005 71.429 golden retriever assistance 4

DHDH006 83.761 chinese crested herding 3

DHDH007 69.236 golden retriever therapy advanced, K99 3

DHDH008 67.035 aussie K99/advanced (obedience, 3 distance work, tricks)

DHDH009 67.593 labradoodle assistance, agility 4

DHDH010 69.324 border collie K99/basic (obedience, distance 2 work, tricks)

DHDH011 72.277 border collie herding, agility 2

DHDH012 70.588 border collie several types of trainings - basic 2 level

DHDH013 67.433 cocker spaniel advanced obedience, touch screen, 3

search

DHDH014 72.719 cocker spaniel basic obedience, touch screen 2

DHDM015 71.196 golden retriever advanced obedience 2

DHDM016 64.640 border collie advanced obedience, touch screen 3

DHDM017 69.939 border collie basic obedience 1

DHDM018 72.411 border collie advanced obedience, herding, 3 frisbee

DHDM019 67.307 border collie advanced obedience, touch screen 3

DHDM020 77.477 border collie advanced obedience, agility, dog 3 dance

*Note.* Face-orientation of training: 1=“*no need to attend to human faces*”, 2=“*some need to attend to human faces*”,

3=“*much need to attend to human faces*”, 4= “*very much need to attend to human faces*”. For dogs with more training types, the number representing the training with highest face-orientation was assigned.

Brain-based cephalic index for each dog was calculated based on its structural image and using the following formula: (brain width [x coordinate distance (leftmost tip of the temporal cortex; rightmost tip of the temporal cortex)]*100) / brain

length [y coordinate distance (frontalmost tip of the olfactory bulb; most posterior tip of the occipital cortex)] .

IDs with the letter “H” after “DHD” are for dogs from the Hungarian site and IDs with the letter “M” after “DHD” are for dogs from the Mexican site.

13
