# Supplementary material for: Comparative Brain Imaging Reveals Analogous and Divergent Patterns of Species and Face Sensitivity in Humans and Dogs
Source: J Neurosci. 2020 Oct 21;40(43):8396–408. doi: 10.1523/JNEUROSCI.2800-19.2020 (PMC7577605; doi:10.1523/JNEUROSCI.2800-19.2020)
Supplement: Figure 2-1 — Visually-responsive regions in the dog and human brain (all conditions vs. baseline). Download Figure 2-1, DOCX file [file ns-JN-RM-2800-19-s06.docx]

Figure 2–1

*Visually-responsive regions in the dog and human brain (all conditions vs. baseline).*

| Cluster *p* | Cluster size |
| --- | --- |
| (FWE-corr) | (voxels) |

Contrast Brain region

Peak T

Coordinates

(x, y, z)

Dogs

| ALL>BL | R mESG | <.001 | 932 | 7.832 | 16, -24, 10 |
| --- | --- | --- | --- | --- | --- |
|  | L cSSG |  |  | 6.270 | -22, -24, 0 |
|  | L rSG |  |  | 5.992 | -18, -12, 12 |
|  | R EMG |  |  | 5.624 | 18, -36, 22 |
|  | L/R MG |  |  | 5.614 | 0, -32, 22 |
|  | L EMG |  |  | 4.671 | -16, -36, 24 |
|  | L rSSG |  |  | 4.372 | -18, 2, 20 |
|  |  |  | Humans |  |  |
| ALL>BL | L FuG | <.001 | 11173 | 22.939 | -34, -58, -18 |
|  | R FuG |  |  | 20.321 | 42, -50, -18 |
|  | R MOG |  |  | 19.838 | 28, -86, 14 |
|  | R FuG |  |  | 19.069 | 24, -80, -10 |
|  | L MOG |  |  | 18.72 | -32, -88, 12 |
|  | L SOG |  |  | 18.328 | -10, -96, 6 |
|  | L FuG |  |  | 18.285 | -28, -76, -14 |
|  | R IOG |  |  | 17.98 | 42, -74, -4 |
|  | R HPC | <.001 | 320 | 18.456 | 20, -30, 0 |
|  | L HPC |  |  | 15.016 | -20, -32, 0 |
|  | L HPC |  |  | 10.002 | -4, -34, -6 |
|  | R IFG | <.001 | 196 | 10.149 | 44, 8, 32 |
|  | R PoCG | <.001 | 33 | 9.078 | 44, -30, 50 |

*Note.* Threshold for reporting for all contrasts was *p*<.001 and cluster *p*<.05. BL=baseline; ALL=all

conditions collapsed; L=left; R=right; mESG=mid ectosylvian gyrus; cSSG=caudal suprasylvian gyrus; rSG=rostral sylvian gyrus; EMG=ectomarginal gyrus; MG=marginal gyrus; rSSG=rostral suprasylvian gyrus; FuG = fusiform gyrus; MOG=middle occipital gyrus; SOG=superior occipital gyrus; IOG=inferior occipital gyrus; HPC=hippocampus; IFG=inferior frontal gyrus; PoCG = postcentral gyrus.

5
