# Supplementary material for: Comparative Brain Imaging Reveals Analogous and Divergent Patterns of Species and Face Sensitivity in Humans and Dogs
Source: J Neurosci. 2020 Oct 21;40(43):8396–408. doi: 10.1523/JNEUROSCI.2800-19.2020 (PMC7577605; doi:10.1523/JNEUROSCI.2800-19.2020)
Supplement: Figure 2-2 — Results of non-parametric random effects analyses of individual binary preference maps within visually-responsive regions in the dog and human brain. Download Figure 2-2, DOCX file [file ns-JN-RM-2800-19-s07.docx]

Figure 2–2

*Results of non-parametric random effects analyses of individual binary preference maps within visually-responsive regions in the dog and human brain.*

Brain region cluster size

(voxels)

Dogs

peak

T

coordinates

(x, y, z)

Conspecific- over face-preference

R mSSG* 21 5.812 16, -24, 12

L mSSG* 14 4.273 -14, -26, 16

L/R SpG 3 3.269 0, -36, 20

Face- over conspecific-preference *no suprathreshold clusters*

Humans

Conspecific- over face-preference *no suprathreshold clusters*

Face- over conspecific-preference

L IOG* 46 14.000 -24, -92, -6

R IOG* 13 9.355 22, -92, -6

R ITG* 45 9.355 44, -48, -24

*Note*. The table includes all clusters with conspecific- over face-preference and face- over conspecific-preference. Threshold for reporting was *p*<.005 and cluster *p*<.05 for dogs and *p*<.0001 and cluster *p*<.001 for humans. * These peaks are also obtained when checked with standard threshold, i.e., p<.001 and cluster p<.05 for dogs and p<.000001 and cluster p<.001 for humans. All peaks ≥16 mm apart are reported. L=left; R=right; mSSG=mid suprasylvian gyrus; SpG=splenial gyrus; IOG=inferior occipital gyrus; ITG=inferior temporal gyrus.

8
