# Supplementary material for: Comparative Brain Imaging Reveals Analogous and Divergent Patterns of Species and Face Sensitivity in Humans and Dogs
Source: J Neurosci. 2020 Oct 21;40(43):8396–408. doi: 10.1523/JNEUROSCI.2800-19.2020 (PMC7577605; doi:10.1523/JNEUROSCI.2800-19.2020)
Supplement: Figure 3-1 — Results from MVPA within visually-responsive regions in the dog and human brain. Download Figure 3-1, DOCX file [file ns-JN-RM-2800-19-s08.docx]

Figure 3–1

*Results from MVPA within visually-responsive regions in the dog and human brain*

| Comparison Brain region | | Cluster *p*  (permutation test, *n*=10,000) | Cluster  size  (voxels) | Mean of  peak accuracy | *SD* of  peak accuracy | Coordinates  (x, y, z) |
| --- | --- | --- | --- | --- | --- | --- |
|  |  |  | Dogs |  |  |  |
| C vs He | L mSSG | <.05 | 67 | .642 | .124 | -15,-25,18 |
|  | R cSSG | <.05 | 40 | .629 | .136 | 21,-25,0 |
|  |  |  | Humans |  |  |  |
| F vs O | R IOG | <.001 | 2201 | .761 | .180 | 38,-84,-2 |
|  | R FuG |  |  | .725 | .158 | 44,-56,-20 |
|  | R IOG |  |  | .711 | .159 | 36,-64,-8 |
|  | R pMTG |  |  | .703 | .148 | 52,-50,8 |
|  | R LiG |  |  | .694 | .169 | 22,-88,-8 |
|  | R FuG |  |  | .692 | .137 | 40,-40,-18 |
|  | R MOG |  |  | .672 | .172 | 26,-90,8 |
|  | R MOG |  |  | .661 | .148 | 40,-76,14 |
|  | R pMTG |  |  | .653 | .139 | 54,-64,0 |
|  | L FuG | <.001 | 1732 | .797 | .148 | -42,-52,-16 |
|  | L IOG |  |  | .747 | .166 | -38,-82,-4 |
|  | L FuG |  |  | .722 | .139 | -44,-68,-16 |
|  | L IOG |  |  | .697 | .139 | -22,-88,-6 |
|  | L pMTG |  |  | .678 | .127 | -50,-66,4 |
|  | L MOG |  |  | .653 | .172 | -30,-92,8 |
|  | R IFG | <.001 | 118 | .672 | .152 | 48,14,30 |
|  | L MOG | <.001 | 91 | .667 | .112 | -28,-88,20 |
| C vs He | R pMTG | <.001 | 158 | .675 | .163 | 46,-64,2 |
|  | R MOG |  |  | .642 | .132 | 42,-80,2 |

*Note.* Threshold for reporting comparisons was *p*<.001 for dogs, *p*<.000001 for humans and cluster *p*<.05 for dogs and

*p*<.001 for humans. All peaks ≥16 mm apart are reported. L=left; R=right; cESG=caudal ectosylvian gyrus; mSSG=mid suprasylvian gyrus; IOG=inferior occipital gyrus; FuG=fusiform gyrus; pMTG=posterior middle temporal gyrus; LiG=lingual gyrus; MOG=middle occipital gyrus; IFG=inferior frontal gyrus

9
