# Supplementary material for: Comparative Brain Imaging Reveals Analogous and Divergent Patterns of Species and Face Sensitivity in Humans and Dogs
Source: J Neurosci. 2020 Oct 21;40(43):8396–408. doi: 10.1523/JNEUROSCI.2800-19.2020 (PMC7577605; doi:10.1523/JNEUROSCI.2800-19.2020)
Supplement: Figure 4-1 — RSA results on dog brain regions with similar activity pattern as select peaks from the human brain. Download Figure 4-1, DOCX file [file ns-JN-RM-2800-19-s09.docx]

Figure 4-1

*RSA results on dog brain regions with similar activity pattern as select peaks from the human brain.*

| Human brain  regions Dog brain regions | | Cluster siz  (voxels) | e  Peak T | Coordinates (x, y, z) |
| --- | --- | --- | --- | --- |
| Direct matching | | | | |
| R AMY | R MG | 17 | 4.237 | 9,-27,24 |
| Functional matching | | | | |
| R AMY | R rESG | 335 | 7.483 | 17,-11,16 |
|  | L cSG | 71 | 5.853 | -17,-11,6 |
|  | L CG | 74 | 5.290 | -5,-7,18 |
|  | R CN | 7 | 4.835 | 7,-1,8 |
|  | R cSSG | 11 | 4.517 | 21,-19,0 |
|  | L PG | 18 | 4.414 | -13,-1,24 |
| R FuG | R MG | 23 | 4.703 | 5,-29,22 |
|  | R mSSG | 22 | 4.586 | 17,-21,18 |

*Note.* Threshold for reporting for all higher-level contrasts was *p*<.001 and cluster *p*<.05. All peaks ≥16 mm apart

are reported. All cluster *p*(permutations *n*=1000)<.001. Two types of models were tested, one focused on response patterns to the same stimuli across species (direct matching) and one on response patterns to the same species- relationships across species (functional matching). L=left; R=right; AMY=amygdala/hippocampus; FuG=fusiform gyrus; MG=marginal gyrus; rESG=rostral ectosylvian gyrus; cSG=caudal sylvian gyrus; CG=cingulate gyrus; CN=caudate nucleus; cSSG=caudal suprasylvian gyrus; PG=postcruciate gyrus; MG=marginal gyrus; mSSG=mid suprasylvian gyrus.

1
