# Supplementary material for: Comparative Brain Imaging Reveals Analogous and Divergent Patterns of Species and Face Sensitivity in Humans and Dogs
Source: J Neurosci. 2020 Oct 21;40(43):8396–408. doi: 10.1523/JNEUROSCI.2800-19.2020 (PMC7577605; doi:10.1523/JNEUROSCI.2800-19.2020)
Supplement: Figure 4-2 — RSA results on dog visually-responsive brain regions with similar activity pattern as select peaks from the human brain. Download Figure 4-2, DOCX file [file ns-JN-RM-2800-19-s10.docx]

Figure 4–2

*RSA results on dog visually-responsive brain regions with similar activity pattern as select peaks from the human brain.*

| Human brain  regions Dog brain regions | Cluster siz  (voxels) | Peak T Coordinates  (x, y, z) |
| --- | --- | --- |
| Functional matc ing | | |

| R FuG | L rESG | 7 | 4.383 | -17,-11,12 |
| --- | --- | --- | --- | --- |
|  | R mSSG | 5 | 4.632 | 17,-21,18 |
|  | R MG | 11 | 4.741 | 5,-29,22 |
| R AMY | R mSSG | 91 | 6.378 | 17,-21,18 |
|  | L cSSG | 5 | 4.732 | -19,-25,6 |
|  | L mESG | 32 | 4.994 | -17,-15,16 |
|  | R EMG | 24 | 4.882 | 11,-27,20 |
|  | L mSSG | 10 | 4.997 | -15,-25,18 |

*Note.* Threshold for reporting for all higher-level contrasts was *p*<.001 and cluster *p*<.05. All peaks ≥16 mm

apart are reported. All cluster *p*(permutations *n*=1000)<.001. L=left; R=right; FuG=fusiform gyrus; AMY=amygdala/hippocampus; rESG=rostral ectosylvian gyrus; mSSG=mid suprasylvian gyrus; MG=marginal gyrus; cSSG=caudal suprasylvian gyrus; mESG=mid ectosylvian gyrus; EMG=ectomarginal gyrus.

1
