# Supplementary material for: Comparative Brain Imaging Reveals Analogous and Divergent Patterns of Species and Face Sensitivity in Humans and Dogs
Source: J Neurosci. 2020 Oct 21;40(43):8396–408. doi: 10.1523/JNEUROSCI.2800-19.2020 (PMC7577605; doi:10.1523/JNEUROSCI.2800-19.2020)
Supplement: Figure 4-3 — Follow-up analyses on effect sizes corresponding to RSA effects observed between visually-responsive dog brain regions and peaks of select human brain regions. Download Figure 4-1, DOCX file [file ns-JN-RM-2800-19-s11.docx]

Figure 4–3

*Follow-up analyses on effect sizes corresponding to RSA effects observed between visually- responsive dog brain regions and peaks of select human brain regions.*

Human brain regions

Dog brain

regions Condition pair

Cohen's

*D*

Effect magnitude

Peak

T

Coordinates

(x, y, z)

R AMY L mSSG CF-HeF .657 medium 3.596 -15,-25,18

R EMG CF-HeF .641 medium 3.514 11,-27,20

L mESG CF-HeF .640 medium 3.505 -17,-15,16

R mSSG CF-HeF .581 medium 3.182 17,-21,18

L cSSG CF-HeF .493 small 2.700 -19,-25,6

L mSSG HeF-HeO .361 small 1.975 -15,-25,18

L cSSG HeF-HeO .330 small 1.807 -19,-25,6

R mSSG CF-CO .310 small 1.700 17,-21,18

L cSSG CF-CO .258 small 1.414 -19,-25,6

L mESG HeF-HeO .230 small 1.260 -17.-15.16

R FuG R MG CF-HeF .656 medium 3.595 5,-29,22

R mSSG HeF-HeO .580 medium 3.176 17,-21,18

L rESG CF-HeF .438 small 2.398 -17,-11,12

R MG CF-CO .420 small 2.298 5,-29,22

R mSSG CF-HeF .412 small 2.254 17,-21,18

L rESG HeF-HeO .395 small 2.164 -17,-11,12

R mSSG HeO-CO .374 small 2.048 17,-21,18

L rESG HeO-CO .344 small 1.885 -17,-11,12

R mSSG CF-CO .268 small 1.467 17,-21,18

*Note.* Cohen’s *D*s are interpreted as follows: .2–.5 is a small effect and .5–.8 is a medium effect (<.2 is a negligible effect and comparisons with negligible-sized effects are not reported). C=conspecific; He=heterospecific; AMY=amygdala/hippocampus; FuG=fusiform gyrus; mSSG=mid suprasylvian gyrus; EMG=ectomarginal gyrus; mESG=mid ectosylvian gyrus; cSSG=caudal suprasylvian gyrus; MG=marginal gyrus.

1
